# Dissecting the regulatory architecture of gene expression QTLs

**DOI:** 10.1186/gb-2012-13-1-r7

**Published:** 2012-01-31

**Authors:** Daniel J Gaffney, Jean-Baptiste Veyrieras, Jacob F Degner, Roger Pique-Regi, Athma A Pai, Gregory E Crawford, Matthew Stephens, Yoav Gilad, Jonathan K Pritchard

**Affiliations:** 1 Department of Human Genetics, University of Chicago, 920 E58th Street, Chicago, IL 60637, USA; 2 Howard Hughes Medical Institute, University of Chicago, 929 East 57th Street, Chicago, IL, 60637, USA; 3 Duke Institute for Genome Sciences and Policy Duke University, 101 Science Drive, Durham, NC 27708, USA; 4 Department of Statistics, University of Chicago, 920 E58th Street, Chicago, IL 60637, USA; 5 Department of Bioinformatics, Wellcome Trust Sanger Institute, Wellcome Trust Genome Campus, Hinxton, Cambridge, CB10 1SA, UK

## Abstract

**Background:**

Expression quantitative trait loci (eQTLs) are likely to play an important role in the genetics of complex traits; however, their functional basis remains poorly understood. Using the HapMap lymphoblastoid cell lines, we combine 1000 Genomes genotypes and an extensive catalogue of human functional elements to investigate the biological mechanisms that eQTLs perturb.

**Results:**

We use a Bayesian hierarchical model to estimate the enrichment of eQTLs in a wide variety of regulatory annotations. We find that approximately 40% of eQTLs occur in open chromatin, and that they are particularly enriched in transcription factor binding sites, suggesting that many directly impact protein-DNA interactions. Analysis of core promoter regions shows that eQTLs also frequently disrupt some known core promoter motifs but, surprisingly, are not enriched in other well-known motifs such as the TATA box. We also show that information from regulatory annotations alone, when weighted by the hierarchical model, can provide a meaningful ranking of the SNPs that are most likely to drive gene expression variation.

**Conclusions:**

Our study demonstrates how regulatory annotation and the association signal derived from eQTL-mapping can be combined into a single framework. We used this approach to further our understanding of the biology that drives human gene expression variation, and of the putatively causal SNPs that underlie it.

## Background

Changes in gene expression are likely to play important roles in adaptive evolution and human disease [1-5]. Much research is focused on understanding exactly how changes in gene expression are encoded at the level of the DNA sequence. One potentially powerful method for dissecting this relationship is by expression quantitative trait locus (eQTL) mapping [6].

Previous eQTL studies have used genetic linkage [7-10] or association analysis [11-19] to identify regions of the genome that contain eQTLs in a variety of different species and cell types. Recent work has shown that eQTLs identified in lymphoblastoid cell lines are also substantially enriched among genome-wide association signals, indicating that many are indeed functionally relevant in primary tissues [20-23].

Previous studies have shown that eQTLs tend to cluster near the transcription start sites (TSSs) of target genes [14,15,17,18]; eQTLs may also be enriched within the transcript regions of the target genes, in exons relative to introns [15], and in conserved regions [24]. However, we still know relatively little about the actual functional context of the SNPs that produce eQTLs, such as the extent to which these tend to occur in active promoter or enhancer regions, in ChIP-seq peaks, or in recognizable transcription factor (TF) binding sites.

One challenge for dissecting the functional basis of eQTLs is that, until now, eQTL mapping in humans has been restricted to incomplete genotype data (for example, phase II HapMap contained approximately 30% of common SNPs [25]). Thus, for most eQTLs, the true causal SNPs were not included in the data sets. Second, while it seems likely that many eQTLs disrupt regulatory elements or motifs, annotation of such features at a genome-wide scale remains difficult. Finally, even with complete sequence data and extensive regulatory annotation, there is usually substantial ambiguity about which site is actually causal for any given eQTL. This is because the causal site is typically in linkage disequilibrium with other nearby tag SNPs and, thus, many non-causal SNPs are also statistically associated with gene expression.

Here we seek to address these three issues using the HapMap lymphoblastoid cell lines as a model system. These cell lines represent a unique resource for our purpose as they have been genotyped at more than 3 million SNPs by the International HapMap Project [25] and many have also been sequenced at low coverage by the 1000 Genomes Consortium [26]. In addition, one of these cell lines is the target of extensive functional characterization by the ENCODE project [27]. In this study, we supplemented available ENCODE data with a large set of experimentally and computationally predicted gene regulatory elements from a variety of other sources. Finally, we dealt with the problem of uncertainty around the causal site using a Bayesian hierarchical model that estimates the enrichment of functional sites within particular types of annotations, while accounting for the uncertainty of which site is causal for any given eQTL [15].

The combination of substantially increased SNP coverage, genome-wide regulatory element annotation and statistical modeling of eQTL location allowed us to make progress towards understanding the functional and sequence context of the genetic variants that drive human gene expression variation at the DNA sequence level. In addition, we show how weighting and combining regulatory annotation data can provide an informative ranking of likely functional SNPs.

## Results

We analyzed gene expression data measured using Illumina WG6 microarrays in 210 HapMap lymphoblastoid cell lines from unrelated individuals, first published by Stranger *et al. *[28]. Compared with other existing data sets, these data include expression measurements for the largest set of individuals that have been resequenced by the 1000 Genomes Consortium and thus provide the greatest power to identify and localize eQTLs. Following expression data cleaning (Materials and methods) we were left with expression measurements on 8,526 genes. Expression normalization and removal of unknown confounders greatly increased our power to detect modest associations [17,29,30] (Figure S1 in Additional file 1). Our genotype data consisted of HapMap genotypes at 3.3 million SNPs for all 210 individuals along with additional genotype calls made by the 1000 Genomes Project for 141 individuals. For SNPs that were called in both the HapMap and 1000 Genomes data, we used the HapMap genotype calls. The genotypes of 1000 Genomes SNPs were imputed in the remaining 69 individuals using BIMBAM [31,32], yielding a total of 13.6 M SNPs per individual. For each of 8,526 expressed genes we tested for eQTLs at all SNPs between 100 kb upstream of the TSS and 100 kb downstream of the transcription end site (nearly all of the compelling signals of eQTLs in this data set lie within this region [14,15]).

In an initial analysis, we used standard linear regression to identify 2,708 eQTLs at a gene-level false discovery rate (FDR) of 1% (corresponding to a *P*-value threshold of *P *= 4 × 10^-6^). Of these eQTLs, 96% were also detected using HapMap SNPs only (at the same *P*-value threshold). However, in many cases, the lowest *P*-value 1000 Genomes SNPs were substantially more significant than the lowest *P*-value HapMap SNPs (791 of the genes have a 1000 Genomes *P*-value at least an order of magnitude smaller than the best HapMap *P*-value (Figures S2A and S3 in Additional file 1)). These observations support the expectation that HapMap SNPs provide good power to detect eQTLs, but frequently miss the functional sites.

In this paper, we will refer to an 'eQTL' as a locus for which at least one SNP shows an association between genotype and gene expression. We assume that each eQTL can be explained by a single causal site, which we will refer to as an 'eQTN' (expression quantitative trait nucleotide). Our primary interest is in understanding the properties of eQTNs. (In this paper we focus on the effects of SNP variation, while recognizing that a modest fraction of eQTLs are caused by other types of variants such as deletions or duplications; see Materials and methods.) In practice, however, there is usually ambiguity as to which SNP is actually driving the observed association. For example, in about 80% of significant eQTLs (at FDR = 1%) there is at least one additional SNP with a *P*-value within a factor of 10 of the most significant *P*-value (Figure S4A in Additional file 1). Moreover, the distance between the significant SNPs for a given eQTL is often tens of kilobases or more (Figure S4B in Additional file 1). This uncertainty poses a serious difficulty for determining whether eQTLs are enriched in any given type of functional element since most functional elements are far smaller than the typical extent of linkage disequilibrium.

### The hierarchical model

To account for this uncertainty, we used a Bayesian hierarchical model, similar to that previously developed by our group [15]. Because it is usually not possible to determine the eQTN for any given eQTL with complete confidence, the hierarchical model instead assigns a posterior probability to each SNP that it is the eQTN and the enrichment estimates are summed over these posterior probabilities. Assigning posterior probabilities allows us to estimate the fraction of eQTNs in an annotation while accounting for uncertainty in determining which SNP is the eQTN.

**Figure 1:**
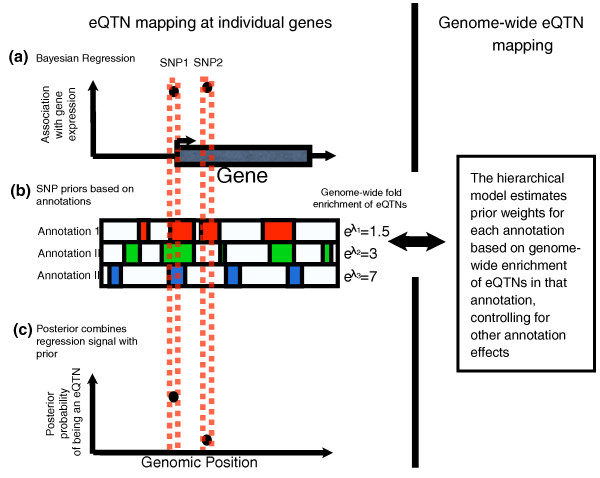
**A schematic outline of the hierarchical model**. **(a) **Two SNPs that are significantly associated with expression level at the adjacent gene (in our method, association is measured using Bayes factors). **(b) **SNP 1 is located in regulatory annotations I, II and III, while SNP 2 is located in regulatory annotation I only. The numbers at the ends of the annotation bars illustrate the fold enrichment of eQTNs in each annotation: these are the exponential of the λl parameters of the hierarchical model. In practice, enrichment levels are estimated using all the genes simultaneously via a hierarchical model. These are combined in a logistic model to estimate the prior probability that any given SNP is an eQTN. **(c) **The hierarchical model assigns a posterior probability that each SNP is an eQTN, combining information from (a, b). Thus, even though the level of association with gene expression is similar for SNPs 1 and 2, more of the posterior probability is assigned to SNP1.

In brief, the model consists of two levels (a cartoon of this is shown in Figure 1). At the level of individual genes, we perform Bayesian regression to test whether the genotypes of each SNP are associated with expression of the gene [33]. The Bayesian regression yields a Bayes factor for each SNP that measures relative support for a model in which that SNP is the eQTN compared to a model in which that gene has no eQTN. We also compute a prior probability that each SNP is the eQTN, based on a variety of annotations (for example, whether the SNP lies within a conserved region or a DNaseI hypersensitive site). The prior probability for each SNP is computed as a logistic function of the SNP's membership in these annotations; the coefficients of the logistic function (denoted by λ_l _for annotation l) are estimated across all genes. By combining the Bayes factors with the prior probabilities we can compute a posterior probability that each SNP is an eQTN for a given gene.

The higher level of the hierarchical model uses all genes with expression data to estimate the coefficients of the logistic prior (that is, the λ_l_). For each annotation, we will refer to the corresponding value of λ_l _as our estimate of the enrichment of eQTNs in that annotation, while controlling for all the other annotations included in the model. eQTN enrichments can be interpreted in the same fashion as a coefficient in a logistic regression. In our case, it is defined as the odds of a SNP being an eQTN given that it is in a certain annotation, divided by the odds if it is not in that annotation, holding all other parameters in the model constant. Estimates of eQTN enrichments are the maximum likelihood estimates of parameters of the hierarchical model. These are computed during the maximization of likelihood of the hierarchical model.

We fit the hierarchical model by maximizing the joint likelihood of the expression data across all genes. This corresponds to setting the λ_l _to their maximum likelihood estimates. At the same time, for each individual eQTL, the posterior probabilities shift towards SNPs that lie in annotations that are enriched for eQTNs in other genes; the amount of shifting of the posterior is weighted by the degree of enrichment of that annotation genome-wide. We have previously shown with simulated data that this approach provides accurate estimates of the genome-wide enrichment of eQTNs within particular features, despite the uncertainty at individual genes [15].

An additional challenge is that both eQTNs and many regulatory annotations are nonrandomly distributed with respect to the TSS and so eQTNs may appear enriched in some annotations by virtue of this spatial distribution alone. We wanted to test whether existing regulatory annotations had explanatory power beyond that expected from their distribution with respect to the TSS. As part of our analysis we therefore developed a background model that captured the effects of distance to the TSS as well as the exon/intron structure of the gene (Materials and methods).

For all annotations discussed in the following sections (DNaseI, histone marks, ChIP-seq, DNaseI foot-prints, core promoter elements and evolutionarily conserved sites) we tested the effect of each annotation separately within the hierarchical model, considering the annotation and the background effects alone. In our final analysis (see 'A combined model of eQTN location' below), we combined all annotations that were significantly enriched in eQTNs, as detected in the first stages of our analysis, in a single model, which we refer to as the combined model. For all analyses using the hierarchical model, we excluded 100 genes with strong eQTLs that we used to test our prior model at the end of the paper (see below for details).

### eQTNs in active chromatin: DNaseI hypersensitivity and histone modifications

DNaseI hypersensitivity and a variety of histone modifications can mark regulatory elements and regions of active transcription or repression [34-38]. We collated publicly available data for eight histone modifications (H3K27ac, H3K4me1, H3K4me2, H3K4me3, H3K9ac, H3K36me3, H3K27me3 and H4K20me1) and DNase-seq data, all collected in HapMap lymphoblastoid cell lines (LCLs). These data were generated by the Bernstein and Crawford groups for the ENCODE project [39,40] and supplemented with additional DNaseI sequencing by our own group [41]. To analyze these data we used the hierarchical model considering each annotation separately.

**Figure 2:**
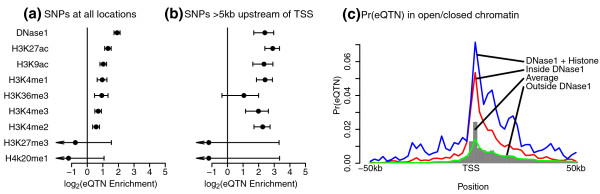
**Estimated fold enrichment of eQTNs in putative regions of active chromatin**. The plots show the enrichment of eQTNs within DNaseI hypersensitive peaks, or within regions marked by a number of histone modifications. **(a) **All locations within the *cis *region around each gene. Error bars show 95% confidence intervals. Arrows indicate that the confidence interval extends beyond the left end of the x-axis. **(b) **Open chromatin 5 to 100 kb upstream of the gene TSS. **(c) **Estimated probability that a random SNP is an eQTN as a function of distance from the TSS (grey bars) or the conditional probability of a random SNP being an eQTN, given that it lies outside or inside a DNaseI hypersensitive site, or within a DNaseI site overlapped by two or more histone marks.

We find that SNPs located within open chromatin, as marked by DNaseI hypersensitivity, are approximately four-fold more likely to be associated with variation in gene expression levels than SNPs outside these regions (Figure 2a,c). Histone marks that have been previously associated with active promoters and enhancers (H3K9ac, H3K4me1, H3K4me2, H3K4me3, H3K27ac) [35-37] are also significantly enriched in eQTNs (Figure 2a; Figure S5 in Additional file 1). In contrast, as might be expected, there is no enrichment for eQTNs in regions marked by the repressive marks H3K27me3 and H4K20me1 (there is instead a weak signal of depletion, albeit nonsignificant, of eQTNs in such regions). The enrichment of eQTNs in regions marked by DNaseI and active histone marks is higher (four- to seven-fold) at distances of > 5 kb upstream of a gene's TSS (Figure 2b; Figure S5 in Additional file 1); the enrichment is strongest for H3K27ac, a modification associated with gene enhancers [35].

Summing over the posterior eQTN probabilities for all eQTLs, we estimate that approximately 20% of all eQTNs occur within DNaseI hypersensitive sites, even though this annotation covers just 1% of the genome. Similarly, over 40% of all eQTNs occur within either a DNaseI hypersensitive site or within a histone-modified region, while this combined annotation covers just 4.5% of the genome (Table S1 in Additional file 1).

### eQTNs and transcription factor binding: ChIP-seq and DNase-seq footprints

Our analysis of regions of open chromatin suggested that a large fraction of eQTNs impact the function of promoters and enhancers, perhaps by modifying protein-DNA interactions that occur in these regions. We next focused on loci of active TF binding identified using two assays: ChIP-seq and DNase-seq footprinting. ChIP-seq identifies fragments of DNA that are bound by a known protein. While ChIP-seq provides binding information for specific proteins of interest, the resolution is somewhat limited as the signal peaks may be hundreds of base pairs in size. In contrast, individual active TF binding sites can be mapped at the motif level by DNase-seq footprinting [41-43]. Here the precise location of TF binding is predicted by identifying DNase-seq 'footprints' detected as protected areas of otherwise hypersensitive regions, which mark the exact location of protein-DNA interaction. DNase-seq footprinting can provide base-pair resolution of the location of factor binding sites; however, there is frequently ambiguity about the active binding factor if multiple factors bind to similar DNA sequence motifs. We used publicly available ChIP-seq data from the ENCODE project for nine TFs [27] as well as DNaseI-based inferences of individual binding sites from the 'Centipede' algorithm [41]. Binding sites were grouped into clusters using sequence similarity (Table S2 in Additional file 1).

**Figure 3:**
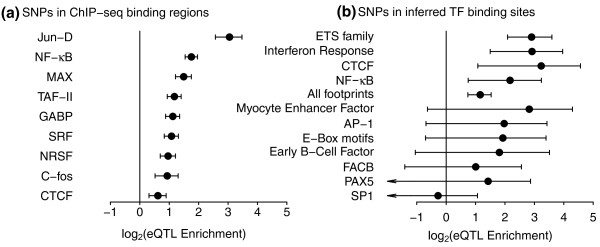
**eQTN enrichments in regulatory elements directly related to transcription factor binding**. **(a, b) **eQTN enrichments in regulatory elements directly related to transcription factor binding as annotated by ChIP-seq (a) or DNase-seq footprinting (b). Of the 26 clusters of DNase-seq footprints tested, 15 had confidence intervals spanning the range (-∞, > 0) and are not shown (Figure S6 in Additional file 1). Error bars show 95% confidence intervals.

Interestingly, our results suggest that many eQTNs influence binding of specific groups of TFs (Figure 3). We find that regions bound by the TF Jun-D are highly enriched for eQTNs (approximately 8.2-fold enrichment above background); strong enrichment is also seen for the immune response factor NF-κB (3.3-fold) (Figure 3a). Our analysis of individual DNase-seq footprints also shows that overall TF binding sites identified using these methods are enriched in eQTNs (2.2-fold; Figure 3a). We also find that specific TFs and groups of TFs are substantially more likely to produce eQTNs. Specifically, we find striking enrichments in binding sites of the ETS family of TFs (approximately 7.5-fold enrichment), interferon stimulated response elements (ISREs; approximately 7.5-fold enrichment), CTCF binding sites (approximately 9.4-fold enrichment) and motifs that bind NF-κB (approximately 4.5-fold enrichment). The most enriched signal is for the ETS TF family of TFs, which are known to be closely involved in B-cell development [44-47]. Other TFs, including the ISRE TFs and NF-κB are key components of the immune response, in particular the cellular response to viral challenge (ISRE, NF-κB, JunD) [48-50].

### eQTNs within the core promoter

**Figure 4:**
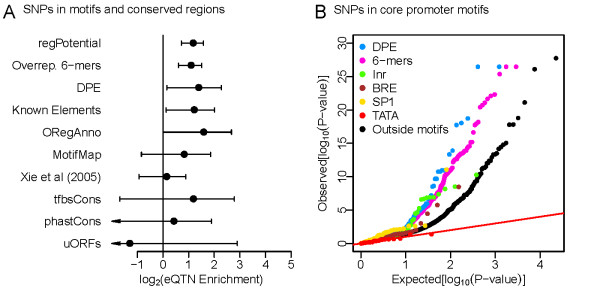
**eQTN enrichment in regulatory elements of the core promoter**. **(a) **The fold enrichments of eQTNs in a variety of predicted regulatory elements based on published methods, sequence motifs and evolutionary conservation. See main text for further details. Only SNPs occurring within 50 bp of the TSS were considered. The confidence intervals for the estimates of enrichment in other core motifs (TATA, SP1, Initiator (Inr) and the TFIIB recognition element (BRE)) were (-∞, > 0) and are not shown. **(b) **The QQ-plots of expected versus observed quantiles of the -log10(*P*-value) for SNPs located in several known core promoter motifs, including the TATA box, the SP1 binding site (or GC-box), the Inr element, the BRE and the downstream promoter element (DPE), as well as in 1,000 6-mer sequences that are highly overrepresented in core promoters.

A large fraction of eQTNs occur very close to the TSS [15], and presumably affect the core and distal promoter architecture. The core promoter is usually defined as the collection of regulatory elements within approximately 50 bp either side of the gene TSS, which serve to position RNA polymerase II correctly [51,52]. We identified individual functional elements in the core promoter using the following computational approaches. We first generated annotations based on known core promoter motifs, such as the TATA box and the initiator (Inr) element. Next we mapped the locations of the 1,000 hexamer words that are most enriched in the core promoter versus the region immediately upstream. Finally, we also identified evolutionarily conserved regions [53], conserved TF binding sites [54,55], known regulatory elements from the literature [56] and upstream ORF-causing mutations [57] within the core promoter region. Our results show that regions of 'high regulatory potential' [58], overrepresented hexamers and the downstream promoter element (DPE) core promoter motif are significantly enriched in eQTNs (Figure 4a). Interestingly, of five known core promoter elements we included here, we find a strong enrichment in only a single motif type, the DPE, with a suggestive but weak enrichment in the Initiator (Inr) motif. DPE has the consensus sequence RGWYV and is typically located 20 to 30 bp downstream of the TSS. Experimental work has suggested that this motif may function as a TATA box in TATA-less *Drosophila *promoters [59]. Perhaps surprisingly, the remaining known core promoter motifs, including the TATA box itself, are not predictive of eQTN location (Figure 4b).

### eQTNs in evolutionarily conserved sites

Evolutionarily conserved regions can often provide valuable information on the location of regulatory elements [60,61]. We obtained phastCons conserved elements [53], phyloP negatively selected sites [62], conserved TF binding sites ('tfbsCons' and 'MotifMap') [54,55] and regions of high 'regulatory potential' [58]. In general, we find that conservation provides surprisingly little information for predicting eQTN location. Only the 'regulatory potential' annotation was marginally significantly enriched in eQTNs (Figure S7 in Additional file 1). We suggest that the relatively small effect of conservation is a result of accounting for a distance from TSS effect in our background model, which may diminish the utility of conservation as an indicator of regulatory elements.

### A combined model of eQTN location

Our survey of existing regulatory annotations identified a number of computational and experimental assays that are significantly enriched in eQTNs. We next assembled these regulatory annotations into a single 'combined' model to reduce uncertainty around putatively causal eQTNs. The annotations included were: DNaseI peaks; the H3K27ac, H3K36me3, K3K4me1, K3K4me2, K3K4me3 and H3K9ac histone marks; known motifs, overrepresented hexamers and high regulatory potential sequences at the core promoter; all the TF ChIP-seq data; and DNase-seq footprints from the ETS, ISRE, CTCF and NF-κB TF groups. In addition to these experimental annotations, we also included our background model, which incorporated distance from the TSS as well as the gene structure.

When parameters are estimated from data, models with a greater number of parameters will always produce a likelihood equal to or greater than a simpler model and so likelihood alone cannot be used to compare combined and background models, which differ in their number of parameters. Instead, we used the Akaike information criterion (AIC), which penalizes models with more parameters. The model with the lowest AIC is the best fit, and a difference of greater than two units of AIC is typically considered significant. Using AIC, our combined model is a significantly better fit to the data than the background model and all the annotation models we used in this study (Figure S8 in Additional file 1). To test for overfitting, we adopted a ten-fold cross validation approach. In cross-validation, because no parameters are estimated from the test data, the likelihood can be directly used to compare these models. In every case the combined model produces a higher likelihood than the background model on the test data set (Figure S9 in Additional file 1). This suggests that our combined model adds significant predictive power beyond the background model.

**Figure 5:**
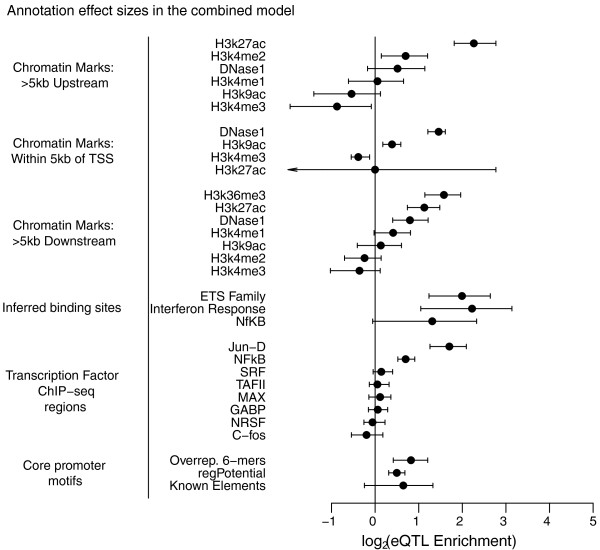
**eQTN enrichments in all functional annotations included in the combined model, ordered by annotation type**. Error bars show 95% confidence intervals. Arrows indicate that the confidence interval extends beyond the left end of the x-axis.

Many of these annotations are correlated and, as a result, their estimated levels of enrichment shrink when included in the same model (Figure 5). This is particularly the case for many of the TF ChIP-seq peaks, only two of which (Jun-D and NF-κB) remain significant when included along with the more generic marks of active chromatin, namely DNaseI and the histone marks. It is also clear that in the combined model, some annotations are substantially more informative than others. For example, in the region > 5kb upstream of the TSS it seems that the best indicator of active regions is the putative enhancer histone mark H3K27ac [35]. The other marks add relatively little when H3K27ac is included in the model, although when tested individually most marks are enriched in eQTNs (Figure S5 in Additional file 1). We note that the correlations between genomic marks will be averaged over by the model, such that the posterior probabilities will accurately reflect the combined effects of all annotations included.

**Figure 6:**
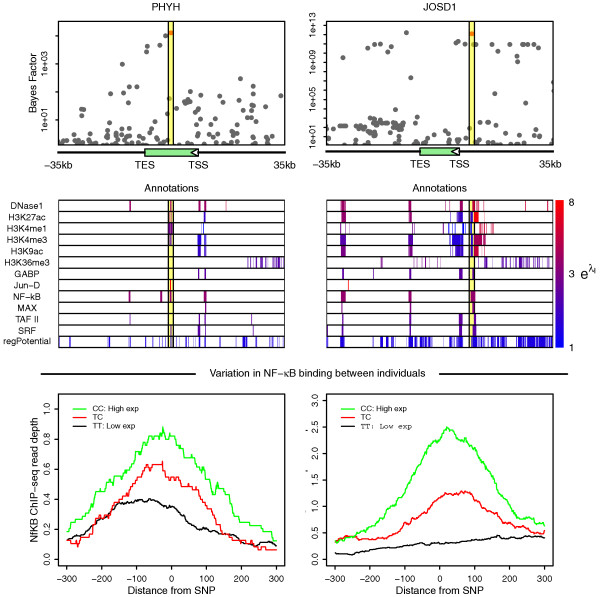
**Examples of two high posterior eQTNs, rs473407 and rs28362527, in two genes**. The first row shows the Bayes factors for each SNP located within a 35-kb window either side of the gene. The second row shows the position, and marginal enrichment level, of some of the regulatory annotations we analyzed here. The positions of the highest posterior SNPs in rows one and two are marked by yellow boxes. Row three shows independent data, not used by the hierarchical model, on the level of NF-κB binding in a 600-bp window centered on each of the two SNPs marked above in yellow, where the ChIP-seq profiles are grouped according to the genotypes at those SNPs. Data on between-individual variation in NF-κB binding were from [63]. TES, transcription end site.

Figure 6 illustrates how the hierarchical model combines information from regulatory annotations with Bayes factors to identify high posterior eQTNs. Here, we selected two example high posterior eQTNs (Pr > 0.5) located in NF-κB ChIP-seq regions (identified using ENCODE data in HapMap individual NA12878). We note that, in this case, we are specifically selecting genes where our model places high weight on an individual SNP being the eQTN. A natural way to identify such SNPs is to select those where the posterior probability is > 0.5 - in other words, our data indicate that this SNP is more likely to be the eQTN for that gene than all other SNPs combined. In both cases, the model selects these SNPs because they are strongly associated with variation in expression and they lie within a number of enriched annotations, including DNaseI hypersensitive regions, multiple histone marks and ChIP-seq peaks.

Independent NF-κB ChIP-seq data from nine individuals [63] are shown in the bottom two panels. These data show that, looking across individuals, the strength of ChIP-seq signal for NF-κB in this region is significantly correlated with the putative eQTN genotypes (*P *= 4.2 × 10^-3 ^and *P *= 3.4 × 10^-4 ^for rs473407 and rs28362527, respectively).

More generally, for high-confidence eQTNs within NF-κB peaks we see a significant enrichment of positive correlation between eQTN genotype and NF-κB read-depth (*P *= 0.013, Kolmogorov-Smirnov test) (Figures S10 and S11 in Additional file 1). For a large fraction of the eQTNs that are significantly correlated with change in binding, the direction of the change is the same as the direction of change in expression (74%; *P *= 6.3 × 10^-4^, sign-test), consistent with the generally accepted role of NF-κB as an activator [64]. Our results therefore suggest that the functions of this group of eQTNs may frequently involve changes in binding level of NF-κB at these locations.

### Prediction of eQTN location using only prior information

The hierarchical model combines regulatory annotations (in the form of a prior model) with the association signal derived from eQTL mapping. We tested the extent to which this prior model (that is, excluding the association signal) places a sensible ranking on which SNPs are most likely to generate eQTLs. Before our analysis, we selected 100 genes with a strong eQTL and for which there was a single strong candidate eQTN SNP. These genes were withheld from all analyses using the hierarchical model. The criteria for selecting these genes were that we required (i) at least one SNP with a *P*-value < 5 × 10^-8 ^(this corresponds to an FDR of 0.01%), and (ii) that the *P*-value difference between the most significant SNP and the next most significant SNP for that gene be at least two orders of magnitude. This *P*-value difference corresponds to requiring that the most associated SNP has a roughly 100-fold higher Bayes factor than any other SNP for that gene.

Simulations indicate that, in the absence of genotyping error, this procedure would correctly identify causal SNPs for > 99% of genes; the corresponding rate with realistic genotyping and imputation error rates is > 90% (Figure S12 in Additional file 1). We may also miss some causal variants (such as structural variants or variants in highly repetitive regions) if they are not included in the SNP data. Note that misidentification of the causal variant will cause our analysis to be somewhat conservative, in the sense that we will tend to underestimate the performance of our prior. These genes will also tend to have lower than average linkage disequilibrium, although this would not seem to have any obvious biasing effect on the performance of the prior.

In the entire data set, 198 genes meet both criteria; the 100 genes that we used were sampled at random from the set of 198 (see Figure S13 in Additional file 1 for examples). We then tested the ability of our prior models to predict the location of the lowest *P*-value SNP. This effectively tests whether the prior can distinguish low *P*-value SNPs using only regulatory annotations, but without information on gene expression variation.

**Figure 7:**
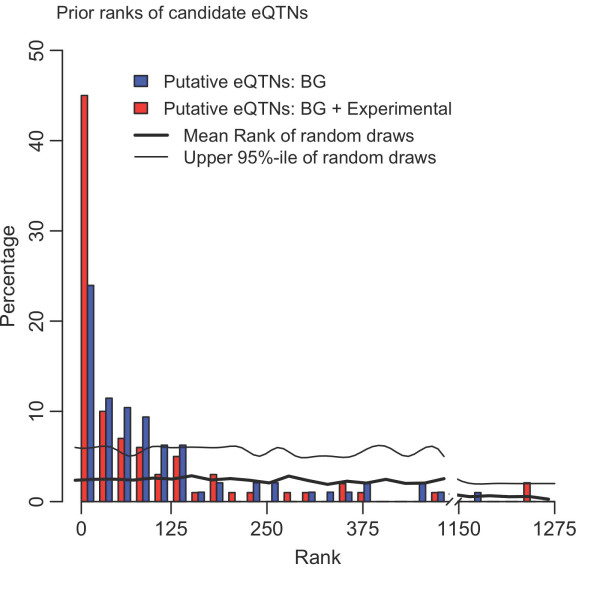
**Prior rankings of SNPs for 100 genes where a single SNP is a clear best candidate for being the 'true' eQTN using the prior probability from the hierarchical model**. The histogram shows the percentage of genes for which the putative causal site is ranked by the prior among the top 1 to 15 SNPs, 15 to 30 SNPs, and so on. Typically, the candidate region for each gene contains approximately 1,200 SNPs. The two prior models correspond to the distance model only (blue) and the distance model plus experimental annotations (red). The 100 genes analyzed here were excluded from all other analyses. BG, background.

For 50% of genes the putative causal site is among the top 3% of SNPs in the genic region based on the prior model, and for a large fraction (70%) the putative causal site is ranked among the top 10% of SNPs in the region (Figure 7). The model with experimental data is significantly better than the distance model alone (*P *= 1 × 10^-5^), and both models are far better than a random prior (*P *< < 10^-16^). Our results suggest that, by itself, regulatory annotation can already provide a meaningful selection of putative eQTNs. Combining this prior with gene expression association signals is therefore a powerful approach for identifying causal variants.

## Discussion

Despite its relevance, the biology underlying human gene expression variation remains poorly understood. To address this problem, we used genome resequencing data from the 1000 Genomes Project to map eQTLs at very high resolution in 210 human LCLs. Our goal was to understand the biological mechanisms disrupted by these variants. We find that a substantial fraction of all eQTNs are located in regions of active chromatin. In addition, active binding sites for immune-related TFs are among the most highly eQTN-enriched regions in the genome. At the TSS, eQTNs appear to frequently disrupt a known core promoter motif but not other well-known elements such as the TATA box. Finally, we showed that eQTNs predicted by our model are also significantly associated with changes in NF-κB binding, and that a combination of regulatory annotations performs well as a prior model of eQTN location.

Open chromatin facilitates the direct interaction of regulatory proteins or complexes with elements in the DNA sequence. A central conclusion of our study is that many eQTNs drive gene expression variation by perturbing this process. In support of this we show that eQTNs are enriched in open chromatin, in DNaseI footprints and in ChIP-seq peaks. One obvious mechanism is that eQTNs may interfere directly with protein-DNA interactions by changing the binding affinity of the DNA for active TFs [39,65].

Aside from altering the binding of TFs, eQTNs may also perturb gene regulation in more subtle ways - for example, by altering the intrinsic nucleosome preferences of the DNA [66]. eQTNs may also act epigenetically by altering the pattern of DNA methylation, with resulting effects on gene expression [67].

The final stages of our analysis suggest that regulatory annotation information, combined in a principled fashion, can identify putatively functional candidate eQTNs. A recent study by Lee *et al. *[24] also addressed the related problems of identifying causal regulators (SNPs) using a regularized regression framework. Interestingly, Lee *et al. *found that evolutionary conservation was more heavily weighted than we observe here. One possibility is that our background model accounts for much of this effect given that conservation is strongly negatively correlated with distance from the TSS [68].

Finally, our work shows that, given a sufficiently large and high resolution training data set, our prior could potentially be used to predict putative regulatory mutations in additional cell lines and tissues. A clear application of this would be the identification of functional candidates from panels of putatively disease- causing SNPs identified in genome-wide association studies. This is of particular relevance given that high-quality data on chromatin structure, DNA methylation and TF binding are now available for a wide variety of cell lines and tissues. Our model provides a straightforward means of integrating these data and is a step towards the goal of uncovering the regulatory architecture underlying human genetic disease and quantitative traits.

## Materials and methods

### Genotype data

Our analysis focused on the 210 unrelated HapMap phase I LCLs studied by Stranger *et al. *[28]. We downloaded 1000 Genomes genotypes for 141 of these individuals, including 44 Yoruba (YRI), 30 unrelated Japanese (JPT), 29 unrelated Han Chinese (CHB) and 43 CEPH (CEU), from the March 2010 SNP release of the 1000 Genomes Consortium [26]. For all HapMap SNPs we used HapMap genotype calls from release 24 of HapMap phase II [25]. We imputed genotypes for the remaining 69 individuals using BIMBAM [31,32], excluding SNPs with a minor allele frequency < 1%. Our final SNP data set consisted of a total of 3.3 million HapMap SNPs and a further 10.3 million 1000 Genomes SNPs. For each gene we considered all SNPs in a window extending 100 kb upstream of the gene TSS and 100 kb downstream of the gene transcription end site.

Our analysis in this paper is restricted to analyzing SNP data, and not other types of variants such as copy number variants or indels, due to the difficulties of incorporating these into our annotation framework. Separate analysis that we have done indicates that these other types of variants account for a small fraction of eQTLs, and hence they introduce little bias into our approach (see [15] for simulations of the hierarchical model with missing variants).

### Expression data pipeline

Expression levels in 210 LCLs were measured on the Illumina WG6 microarray in four HapMap populations, as described in [28]. We remapped the probes from the array to build 36 (hg18) of the human genome using MAQ [69], selecting only those probes that matched a single unique location with zero mismatches. Of the 47,296 probes on the array, 41,729 fulfilled these criteria. We next selected only those probes that overlapped an annotated exon or exon-exon boundary, as defined in ENSEMBL release 52 (March 2009). We found that 18,414 probes mapped to known exons, which target a total of 15,757 genes. Of these, 10,131 probes overlapped one or more SNPs in our data. To remove effects of these SNPs on probe hybridization, for each probe we regressed expression level on the genotype of the SNP located within the probe. In 2,122 cases this regression was significant (*P *< 0.05) and we used the residual of the regression as the expression measurement.

High-dimensional expression data sets are frequently affected by a variety of unknown confounding factors that can induce large-scale dependencies between gene expression levels. Such dependencies can reduce power and induce spurious associations in the data [29,30,70,71]. Following a similar strategy to that outlined in [17], we calculated the first 26 principal components (PCs) of the expression data matrix, after centering the data within each individual. The number of PCs was determined by first calculating PCs of 100 data sets in which expression levels were permuted with respect to individual, to create a distribution of variance explained under the null hypothesis of no "batch" effects on gene expression. Using this null distribution, we calculated an empirical *P*-value for each of the first 30 PCs, and selected only those with *P *< 0.05, leaving the first 26. The expression level of each gene was fitted in a linear regression model with population, sex and the 26 PC scores as potential covariates. The optimal number of PCs to include as covariates in each gene model was selected by elastic net regression [72]. The tuning parameters were selected by leave-one-out cross-validation. The residuals of this regression for each gene were set to the quantiles of a standard normal, separately for each population. Thus, the expression phenotypes in our analysis were the quantile-normalized residuals of expression level after regressing out effects of population, sex and up to 26 PCs. Removal of PCs substantially increased the number of eQTLs we detected in our data set, when compared with a data set with no PCs removed (Figure S1 in Additional file 1), and to our previous study [15]. The implementation of the elastic net regression provided relatively slight improvement over the analysis with 26 PCs removed for all genes. Our expression data pipeline attempts to deal with the possible effects of population structure and expression heterogeneity, which can have a substantial impact on power to detect eQTLs [29,71].

We restricted our analysis to those genes that were expressed in LCLs, where we defined expressed genes using RNA-seq data from a separate analysis in our lab [17]. A gene was defined as expressed if the normalized number of reads per site was greater than 10^-10 ^in over half the individuals (71) in the RNA-seq data set (mean number of reads per lane, 5.35 × 10^6^, read length 35 and 46 bp). This arbitrary threshold was set by visual inspection of the distribution of the normalized number of reads per site across all genes and all individuals. We removed a total of 7,231 genes that had low or zero expression by this definition, leaving a total of 8,526 genes for analysis.

### Modeling and analysis

#### Linear regression

In our initial analysis we used standard linear regression to detect associations with expression, using the same model as in our Bayesian regression analysis. The gene-level FDR was computed by permuting the expression data with respect to the individuals, 100 times, and regressing the expression data on genotype in each of the permuted data sets. This allowed us to estimate the number of associations observed under the null hypothesis of no relationship between genotype and gene expression level [15].

#### The hierarchical model

The complete details of the hierarchical model are provided in Additional file 1 (Supplementary methods). Briefly, the hierarchical model is based on a Bayesian approach to inferring genotype-trait association, as described in [33]. Bayes factors are used as components of a mixture model to describe the observed expression data:

$${\text{L(Y}}_{\text{k}}|\Theta \text{) = }\Pi_{0}{\text{P}}_{\text{k}}^{0}+\left(1-\Pi_{0}\right){\text{P}}_{\text{k}}^{1}$$

where Θ are the model parameters, Π_0 _is the probability that a gene does not have an eQTL, ${\text{P}}_{\text{k}}^{0}$ is the conditional probability of the observed expression data given that there is no eQTL, and ${\text{P}}_{\text{k}}^{1}$ is the conditional probability of the expression data given there is a single eQTN. Here:

$${\text{P}}_{\text{k}}^{1}=\sum_{\left(\text{j}=1\right)}^{\text{Mk}}\pi_{\text{jk}}$$

where π_jk _is the prior probability that SNP j is the eQTN, ${\text{P}}_{\text{k}}^{1}$ is the conditional probability of the data, given that SNP j is the eQTN, and Mk is the number of SNPs in the candidate region of gene k. Prior data, in the form of regulatory annotations, are included using a logistic link function:

$$\pi_{\text{jk}}=\exp\left({\text{x}}_{\text{jk}}\right)/\sum_{\text{j}\prime }^{\text{Mk}}\text{exp(}{\text{x}}_{\text{j}\prime \text{k}}\text{)}$$

where:

$${\text{x}}_{\text{jk}}=\lambda_{1}\delta_{\text{jk}1}+\ldots +\lambda_{1}\delta_{\text{jkl}}$$

The λ_l _represent the additive effect of annotation l on the log-odds of a single SNP being an eQTN and the δ_jkl _are indicator variables such that δ_jkl _= 1 if a SNP is located inside annotation l, and 0 otherwise.

### Annotations

#### DNase-seq, ChIP-seq for transcription factor binding and histone modifications

We generated DNase-seq data in our own group from two additional cell lines (NA18507 and NA19239). Raw reads from these experiments were mapped to the genome using BWA [73]. We removed reads that mapped to more than one location in the genome, had a gapped alignment and/or more than two mismatches to the reference genome. To call enriched regions we implemented a simple sliding window. For each 150-bp window we counted the number of reads overlapping each site, and obtained a smoothed window average using a Gaussian kernel (bandwidth of 50 bp). We set a window threshold, based on Monte-Carlo simulation such that the estimated FDR of our threshold, under a null hypothesis of randomly distributed reads in the genome, was < 1 × 10^-6^. ChIP-seq tags typically target the ends of ChIP fragments rather than the center [74], and because of this, reads targeting modified histones have a strand-specific bias in location - namely, reads on the '+' strand are located 5' of the cross-linked protein-DNA fragment, while reads on the '-' strand are located 3' of the fragment. We implemented a strand-specific correction by shifting the position of reads mapping to the '+' strand 73 bp 3' and reads on the '-' strand 73 bp 5'. Finally, we downloaded publicly available ChIP-seq data from the ENCODE project (generated by the Bernstein, Myers and Snyder groups) for the following TFs: CTCF, C-fos, GABP, Jun-D, Max, NRSF, SRF, and TAFII. Peak locations for each TF were derived using MACS [74] with the default parameter settings.

#### Evolutionary conservation and literature-derived elements

PhastCons conserved elements [53], PhyloP scores [62], putatively conserved TF binding sites (tfbsCons), regulatory potential (RP) scores [58] and the literature-derived regulatory elements in the OReGanno database [56] were downloaded from the UCSC genome browser [54]. We removed all phastCons elements mapping to known protein-coding exons prior to their inclusion in the model and annotated all regions that had a regulatory potential score of > 0.1 as putatively regulatory.

#### The core promoter

Known core promoter motifs were selected from the literature [52] and mapped to the region ± 50 bp of the TSS of each gene. These elements included the TATA-box (TATAAA), the GC-box or SP1 binding site (CCCCGCCCCG), the TFIIB recognition element (BRE; SSRCGCC) and the DPE (RGWYV). To define overrepresented words, we compared the frequency of hexamers in the region ± 50 bp of the TSS with a control region (-100 to 50 bp upstream of the TSS). A word was defined as overrepresented if its observed frequency in the core promoter differed significantly from that in the control region, by binomial test. We selected the top 1,000 hexamers from this test (*P *< 10^-10^) and mapped locations of all occurrences of these words within the core promoter region. Upstream ORF mutations were identified from [57].

#### DNaseI footprints

DNaseI footprints were taken from a previous study of TF binding sites in LCLs [41]. Footprints were divided into clusters based on the positional overlap of predicted bound regions. Only clusters for which the total length of annotated sequence (that is, concatenated sites) exceeded 100 kb of annotated sites were included in our analysis. Footprints can be obtained from [75].

#### Simulation of causal eQTLs

We used Monte-Carlo simulations to determine whether the criteria we used to select relatively unambiguous 'causal' eQTNs were appropriate (namely, that the eQTL should have at least one SNP with *P *< 5 × 10^-8 ^and a minimum difference of two orders of magnitude in *P*-value of the best and next best SNP). For each simulated replicate we randomly drew a gene from the original list of 15,757 genes. We then randomly defined a single SNP as causal. Expression data were simulated for that eQTN according to the linear model outlined in the 'Bayesian regression' section of the Supplementary methods in Additional file 1. The eQTN effect size was simulated as a random draw from the mixture of normal distributions outlined in the Supplementary methods in Additional file 1. The probability of drawing from a given distribution was estimated by the hierarchical model. Next, for each individual we simulated random normally distributed error around the genotype mean. The variance of the error term was estimated from the residuals of the linear regressions. We also investigated the impact of genotyping error by randomly changing a fraction of all genotypes according to the stated genotype error rates of the HapMap SNPs (0.5%) [25] or the 1000 Genomes SNPs (1 to 3%) [26]. Finally, for a variety of thresholds we asked how often a given set of criteria resulted in selection of a non-causal SNP as causal.

#### Variation in NF-κB binding

We downloaded smoothed estimates of NF-κB ChIP-seq read depth in ten LCLs from the ENCODE project [27,65]. Smoothed estimates were normalized by the total number of reads in each lane. We identified 397 high-posterior eQTNs that also lay in NF-κB ChIP-seq peaks identified in NA12878 and for which at least two of the three genotypes were observed the ten individuals analyzed in [65]. For each candidate eQTN we regressed the read depth at the eQTN on genotype.

#### Data availability

All eQTLs and high-posterior eQTNs detected are available from the eQTL browser at http://eqtl.uchicago.edu/. The source code to fit the hierarchical model, our full data set and parameter estimates are available at: http://eqtnminer.sourceforge.net/.

## Abbreviations

AIC: Akaike information criterion; bp: base pair; ChIP-seq: chromatin immunoprecipitation coupled with high-throughput sequencing; DPE: downstream promoter element; eQTL: expression quantitative trait locus; eQTN: expression quantitative trait nucleotide; FDR: false discovery rate; ISRE: interferon stimulated response element; LCL: lymphoblastoid cell line; NF: nuclear factor; ORF: open reading frame; PC: principal component; SNP: single nucleotide polymorphism; TF: transcription factor; TSS: transcription start site.

## Supplementary Material

Additional file 1 **Additional material**. Contains all supplementary tables and figures, as well as supplementary methods. Click here for file
