# Supplementary material for: Dissecting the regulatory architecture of gene expression QTLs
Source: Genome Biol. 2012 Jan 31;13(1):R7. doi: 10.1186/gb-2012-13-1-r7 (PMC3334587; doi:10.1186/gb-2012-13-1-r7)
Supplement: Additional file 1 — Additional material. Contains all supplementary tables and figures, as well as supplementary methods. [file gb-2012-13-1-r7-S1.PDF]

Dissecting the regulatory architecture of gene expression QTLs

Supplementary Methods

Here we describe the details of our analysis using the hierarchical model.

Linear Regression

In our initial analysis we used standard linear regression to detect associations with expression, using the same model as in our Bayesian regression analysis (Eqn. 1). The gene-level FDR was computed by permuting the expression data with respect to the individuals, 100 times, and regressing the expression data on genotype in each of the permuted data sets. This allowed us to estimate the number of associations detected observed under the null hypothesis of no relationship between genotype and gene expression level [1].

Bayesian Regression

Our hierarchical model is based on a Bayesian approach to inferring genotype-trait association, as described in [2], in that we use a Bayes Factor (BF), rather than a P-value, to assess the evidence that a given SNP is an eQTL for a given gene. We start by assuming a simple linear model of gene expression:

$$y_{ik} = \mu + a_j g_{ij} + \epsilon_{ijk} \tag{1}$$

where y_{ik} is the expression level of gene k in individual i, μ is the mean expression level of individuals who are homozygous for the major allele, a is the additive effect of the minor allele on expression level, g_{ij} is number of minor alleles at SNP j in individual i and ϵ_{ijk} is a random normally-distributed error term. Previous work has suggested that, in these data at least, the effects of dominance are small, relative to additive effects, and we do not model them here [1]. Following [1] we assume that, when SNP j is an eQTN, the a_j are drawn from a mixture of five normal distributions with mean 0

and standard deviation, $\sigma_a \in (0.05, 0.1, 0.2, 0.4, 0.8, 1.6)$.

For a SNP j in the candidate region of gene k we now define two competing hypotheses to explain the observed expression data: H_0, gene k does not have an eQTL and H_{1jk}, that SNP j is the functional nucleotide that produces the eQTL signal (in our notation, the eQTN) for gene k. Specifying a prior distribution on the effect sizes allows the ratio of probabilities of these two hypotheses to be computed analytically (Equation 13 of [2]). This ratio is known as a Bayes Factor (BF) and is defined as:

$$BF_{jk} = P^1_{jk}/P^0_k \tag{2}$$

where $P^1_{jk} = Pr(Y_k|H_{1jk})$, $P^0_k = Pr(Y_k|H_0)$ and Y_k is the observed expression data in gene k. The Bayes Factor is a central component of the hierarchical model because it measures support for a clearly stated alternative hypothesis relative to the null. It is the probability of this alternative that we are explicitly trying to model.

The hierarchical model

In what follows, we make a explicit distinction between an eQTL (a signal of association between genotype and gene expression phenotype) and eQTN (the specific functional nucleotide underlying the eQTL). Consider gene k with a surrounding *cis* "candidate" region, containing M_k SNPs. The likelihood of the observed expression data (Y_k), can be written as a mixture of two components:

$$L(Y_k|\Theta) = \Pi_0 P^0_k + (1 - \Pi_0)P^1_k \tag{3}$$

where Θ are the model parameters, Π_0 is the probability that a gene does not have an eQTL, P^0_k is the conditional probability of the observed expression data given that there is no eQTL and P^1_k is the conditional probability of the expression data given there is a single eQTN. P^1_k can be decomposed

into individual contributions from each SNP as follows:

$$P_k^1 = \sum_j^{M_k} \pi_{jk} P_{jk}^1 = \sum_j^{M_k} \pi_{jk} BF_{jk} P_k^0 \tag{4}$$

where π_{jk} is the prior probability that SNP j is the eQTN (given that there is an eQTN) and P_{jk}^1 is the conditional probability of the data, given that SNP j is the eQTN.

The likelihood of a single gene's expression can now be written:

$$L(Y_k|\Theta) = P_k^0(\Pi_0 + (1 - \Pi_0) \sum_j^{M} \pi_{jk} BF_{jk}) \tag{5}$$

Empirical Bayesian Prior Probability of a SNP being an eQTL

The framework outlined above allows a prior to be put on every SNP in the *cis* region around gene k. To include prior information, such as location or experimental annotations, we model the SNP prior π_{jk} using a logistic function:

$$\pi_{jk} = \frac{\exp(x_{jk})}{\sum_{j'}^{M_k} \exp(x_{j'k})} \tag{6}$$

where:

$$x_{jk} = \lambda_1 \delta_{jk1} + ... + \lambda_l \delta_{jkl} \tag{7}$$

The λ_l represent the additive effect of annotation l on the log-odds of a single SNP being an eQTN and the δ_{jkl} are indicator variables such that δ_{jkl}=1 if a SNP is located inside annotation l, and 0 otherwise. Equation (6) is also known as a "softmax activation function" and is a generalisation of a logistic function to multiple variables, such that the sum of the $\pi_{jk} = 1$. This reflects the underlying assumption of our model that there is a single eQTN per gene. There are two additional points to consider. The first is that the λ_l are assumed to be common to all genes - this is the hierarchical aspect of our model. Secondly, the λ_l are estimated from the data and so the prior on each SNP is also "learned", rather than specified before the experiment. Thus, our method is classified as "empirical" rather than truly Bayesian. A key advantage of this approach is that a decision on

whether experimental information should be included in the prior is informed by the data: i.e. by their ability to predict eQTN location.

Partitioning the prior

The model outlined above computes the prior on every SNP from the same set of L annotations. However, a SNP may influence gene expression via multiple biological pathways and is unlikely to perturb each with equal probability (e.g., a SNP which removes a donor splice site is highly likely to influence splicing, but not transcription, while the opposite may be true for a SNP inside a TATA box next to the TSS). This heterogeneity can lead to inconsistencies when all annotations are considered jointly. For example, while it may be reasonable that the probability with which a SNP affects transcription is related to distance to the transcription start site, this seems inappropriate for SNPs that affect splicing. A more biologically-motivated approach is to partition the set of L annotations into N components which are likely to affect similar biological mechanisms. The prior, π_{cjk} that a SNP j affects expression of gene k via the mechanism represented by component c is identical to equation (6), but is now computed only for the subset of annotations L_c in component c. The total prior on SNP j can now be written:

$$\pi_{jk} = \sum_{c}^{N} \Pi_c \pi_{cjk} \tag{8}$$

where Π_c is the conditional prior probability that a SNP affects component c, given that it is an eQTN. The Π_cs are also considered parameters of the model. In this framework, groups of annotations can be considered independently from one another and effects such as distance to the TSS can be appropriately confined. The total likelihood of the expression data in gene k is now:

$$L(Y_k|\Theta) = P_k^0(\Pi_0 + (1 - \Pi_0) \sum_{c}^{N} \sum_{j}^{M_c} \Pi_c \pi_{cjk} BF_{jk}) \tag{9}$$

where M_c denotes the number of SNPs in the annotations of component c.

A three-component model: transcription, gene structure and probe exon

In our prior model, we consider three distinct pathways by which a SNP can affect gene expression. In the first component, Π_1, we group all SNPs which may affect transcription rate. All experimental annotations we focused on in this study were included in this component. Additionally, in this component we allowed the prior to be affected by distance to the TSS. We modelled distance to TSS as a discrete effect, where SNPs were assigned to distance bins of between 10, 5 and 2.5kb in size up to a maximum of 100kb upstream and 200kb downstream of the TSS. All SNPs will get some contribution to their prior from Π_1. The second component, Π_2, models the effect of SNPs located within a coding exon, $5'$ or $3'$UTR or intronic splice site. Finally the third component, Π_3, models the effect of SNPs on the expression level of the exon which contains the microarray probe (the probe-exon). Here we include those sites which may affect differential expression or splicing of the probe-exon, which will manifest as differences in gene expression. Only SNPs within the probe exon or the splice sites surrounding it get a contribution to their prior from Π_3. In this study we focus solely on results from component Π_1, although we incorporated the additional effects of gene structure which we have previously found to be enriched in eQTL [1].

Likelihood maximization

The following log-likelihood function was maximized with respect to the parameters across all K genes in our data:

$$\log L(Y_k|\Theta) = \sum_{k=1}^{K} \log(P_k^0) + \sum_{k=1}^{K} \log\left(\Pi_0 + (1-\Pi_0)\sum_{c}^{N}\sum_{j}^{M_c} \Pi_c \pi_{cjk} BF_{jk}\right) \tag{10}$$

We used a golden section search to maximise each parameter in succession, over multiple iterations. For all models fit, visual inspection of the parameter estimates and log likelihoods suggested that convergence was typically reached after 10 iterations. As in [1], the λ_l were initialised by setting $\Pi_0 = 0$, while the Π_c were set to $1/N$.

Posterior probability that SNP j is an eQTN

Recalling that H_{1jk} is the hypothesis that SNP j is the eQTN for gene k, the posterior probability, $P(H_{1jk}|Y_k, \hat{\Theta})$ can be computed using Bayes' rule.

$$P(H_{1jk}|Y_k) = \frac{P(Y_k|H_{1jk})P(H_{1jk})}{P(Y_k)} \tag{11}$$

Using the "hat" notation to denote parameters which are estimated from the data:

$$\begin{aligned} P(Y_k|H_{1jk}) &= BF_{jk}P_k^0 \\ P(H_{1jk}) &= (1-\hat{\Pi}_0)\sum_c^N \hat{\pi}_{cjk} \\ P(Y_k) &= P_k^0(\hat{\Pi}_0 + (1-\hat{\Pi}_0)\sum_c^N \sum_j^{M_c} \hat{\pi}_{cjk}BF_{jk}) \end{aligned}$$

and so:

$$P(H_{1jk}|Y_k) = \frac{BF_{jk}(1-\hat{\Pi}_0)\sum_c^N \pi_{cjk}}{\hat{\Pi}_0 + (1-\hat{\Pi}_0)\sum_c^N \sum_j^{M_c} \hat{\pi}_{cjk}BF_{jk}} \tag{12}$$

Cross-validation

We performed 10-fold cross-validation to test the robustness of our combined model. We divided our list of genes into 10 random groups. We created 10 paired test and training sets, such that each 1/10th of the data is represented exactly once in each of the test sets, and the corresponding training set includes the remaining 9 groups. We then fit two models to each of the training sets: a baseline or background model (the distance model only) and our model with functional annotations (distance + functional annotations). We then used the parameters estimated on the training set to compute the likelihood of the held-out test set for both the background model and the functional annotation model. For all ten test sets, the functional model had improved fit compared to the background model (mean of 18 units of likelihood), indicating that the functional model provides a substantially better fit to the data (Supplementary Figure S9).

Supplementary Figures

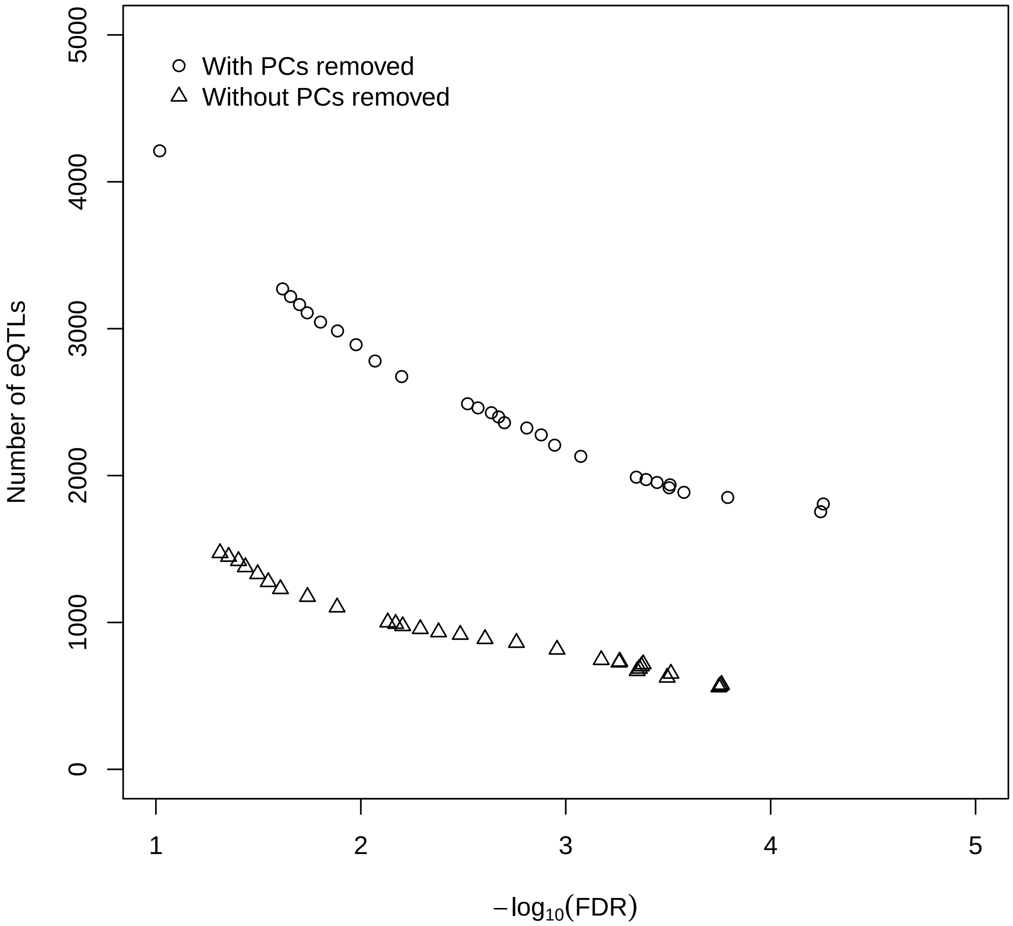

Figure S1: Number of eQTLs (defined as the number of genes where the lowest P-value SNP is less than a given FDR cutoff) by FDR rate for data sets with and without principal components removed.

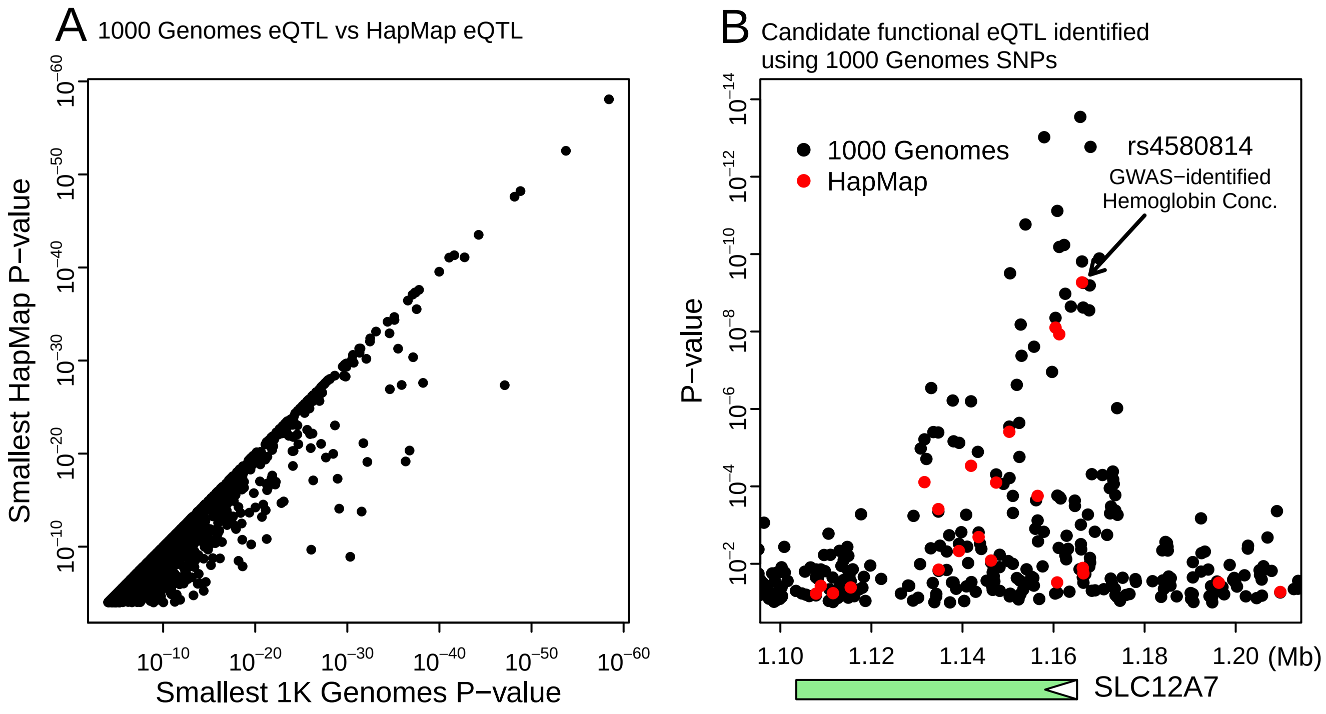

Figure S2: Comparison of eQTL mapping with the HapMap and 1000 Genomes SNPs. Panel A shows a comparison of the smallest p-values obtained using the union of the 1000 Genomes and HapMap SNPs (x-axis) versus the HapMap SNPs alone (y-axis). Each dot represents a single gene with at least one SNP significant at p$< 4 \times 10^{-6}$ (FDR=1%). Panel B shows an example of improved association signal using the 1000 Genomes SNPs in the vicinity of the SLC12A7 gene. The x-axis represents physical position on chromosome 5 in Mb. In addition to being associated with gene expression, SNP rs4580814 has also been previously identified in a genome-wide association study of mean hemoglobin concentration in a Japanese population [3].

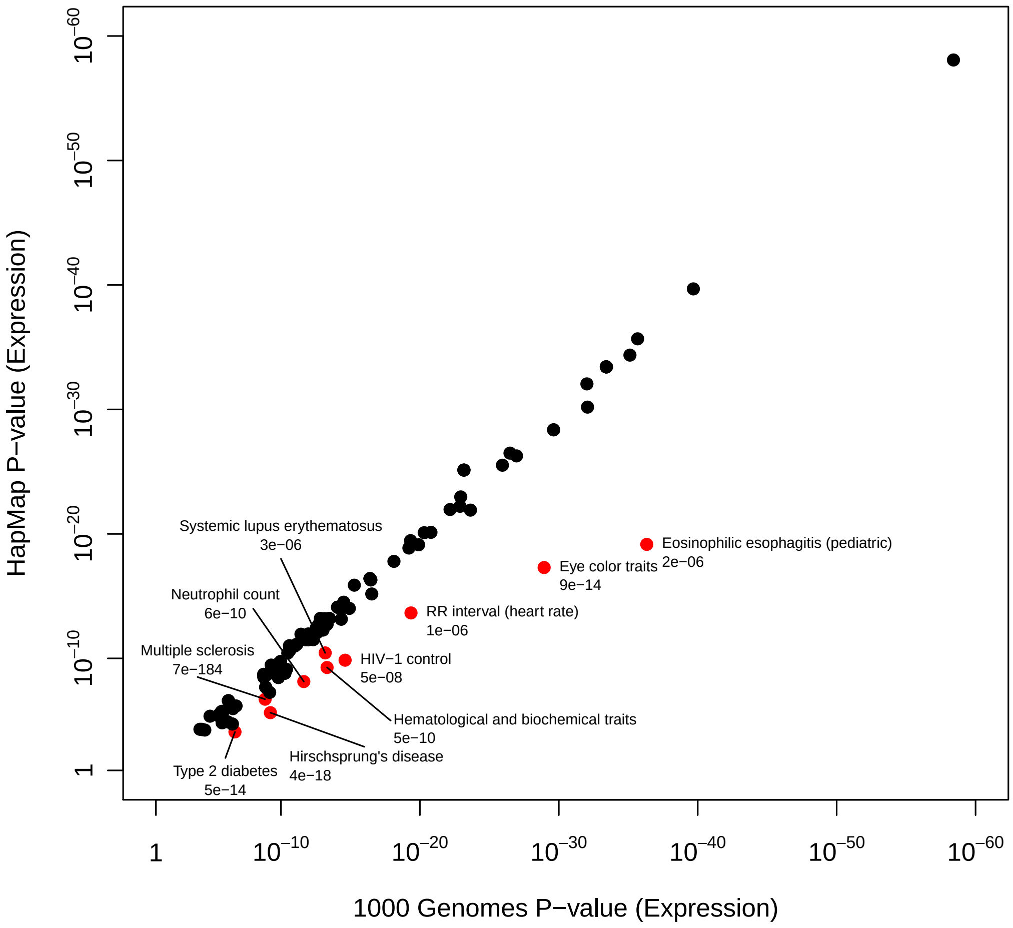

Figure S3: Comparison of eQTL mapping with the HapMap and 1000 Genomes SNPs. The plot shows p-values for the union of the 1000 Genomes and HapMap SNPs (x-axis) versus the HapMap SNPs alone (y-axis) for SNPs that are in linkage disequilibrium with phenotype-associated SNPs identified in genome-wide association studies. Red points denote SNPs where a previously hidden 1000 Genomes SNP has a substantially lower (1 order of magnitude) p-value than the lowest p-value HapMap SNP. The list of GWAS SNPs, and their associated traits, were from [4]

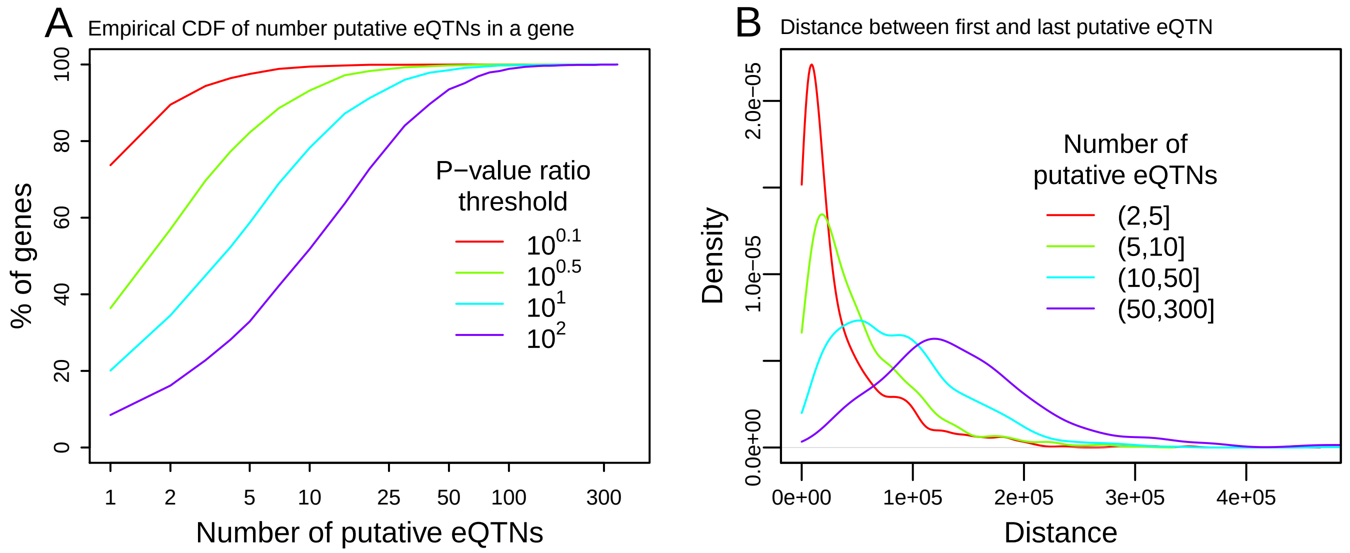

Figure S4: Uncertainty in identifying the causal eQTNs using a p-value criterion, for 2708 genes that had an eQTL at FDR=0.01. Panel A shows the cumulative distribution of the numbers of putative eQTNs in a gene, where SNPs were counted as putative eQTNs when the ratio between their p-value and the lowest p-value SNP for the gene was less than a given power of 10, shown in the legend. Panel B shows the distance between the first and last SNP, of all SNPs where the ratio between their p-value and the lowest p-value SNP for the gene was less than 100.

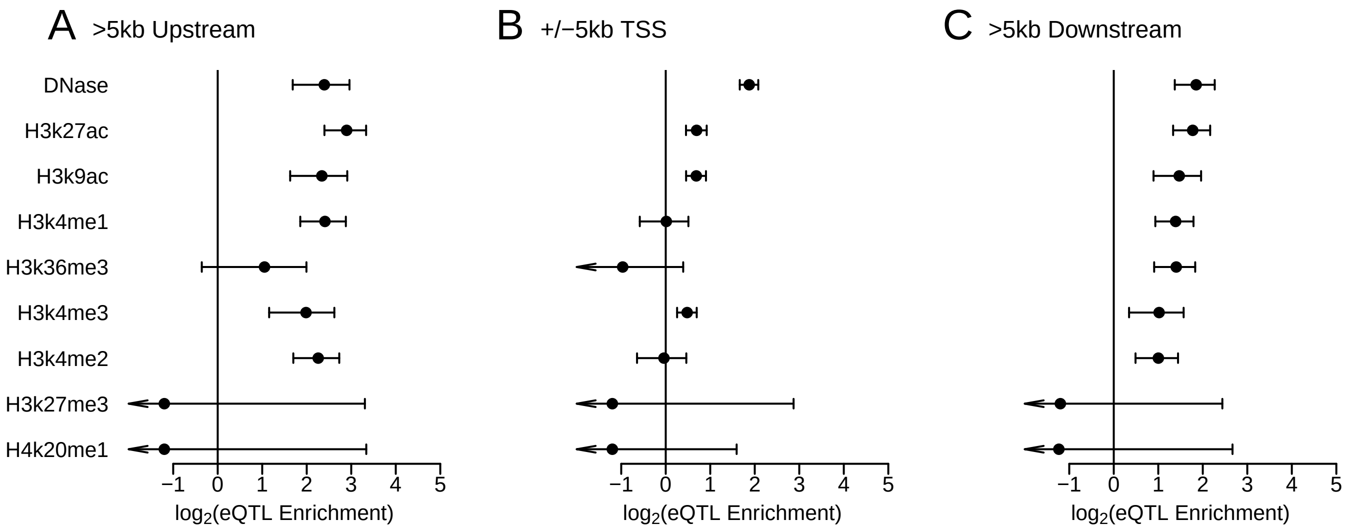

Figure S5: Fold enrichment of eQTN in open chromatin by location. Panel A, B and C show the enrichment in eQTN within DNase1 hypersensitive peaks, or within regions marked by various histone modifications 5kb upstream of the TSS, within 5kb of the TSS and 5kb downstream of the TSS respectively. Error bars show 95% confidence intervals. Arrows denote a C.I. lower bound below than the scale of the x-axis.

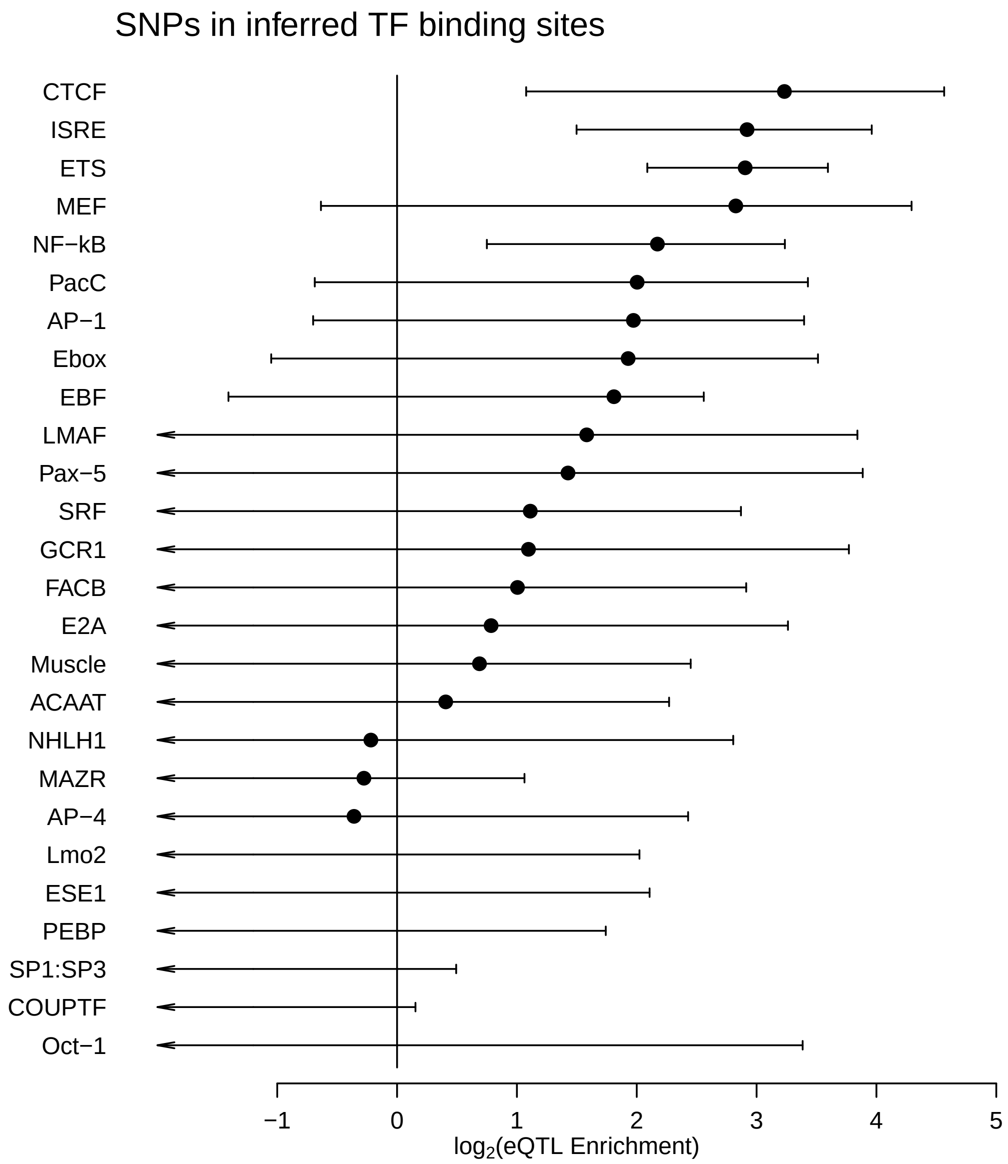

Figure S6: eQTN enrichments in inferred transcription factor binding sites for all 26 clusters annotated by DNase1 hypersensitivity footprinting. Error bars show 95% confidence intervals. Arrows denote a C.I. lower bound below than the scale of the x-axis.

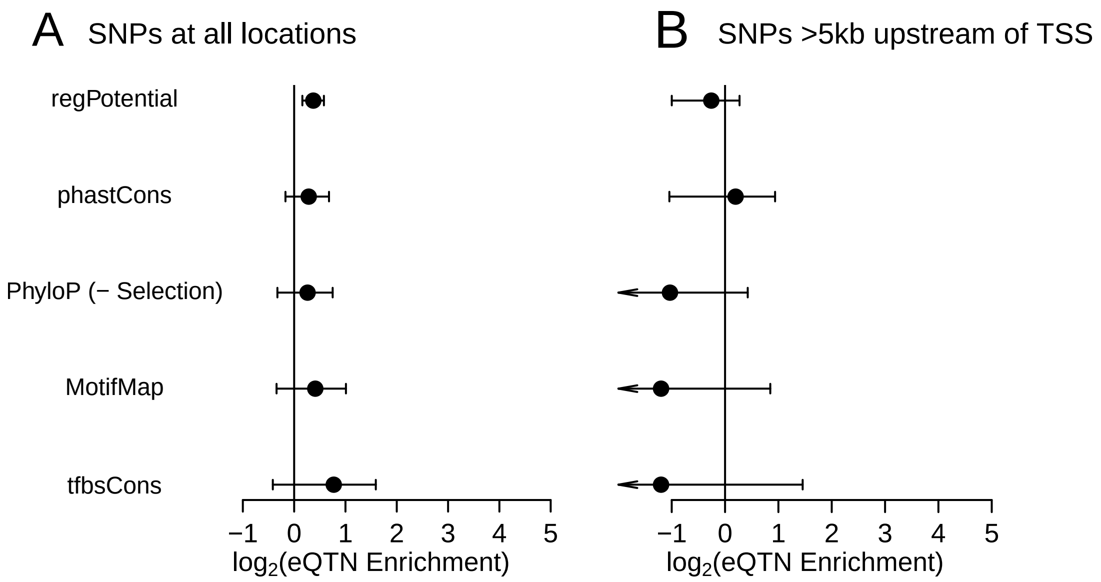

Figure S7: Enrichment of eQTNs at evolutionarily conserved sites. Enrichments are shown for all sites (Panel A) and for sites >5kb upstream of the TSS (Panel B). The annotations are for sites with high regulatory potential [5], in highly conserved PhastCons elements [6], in negatively selected sites from PhyloP [7] and in conserved transcription factor binding sites from the tfbsCons UCSC track and MotifMap [8]. Error bars show 95% confidence intervals.

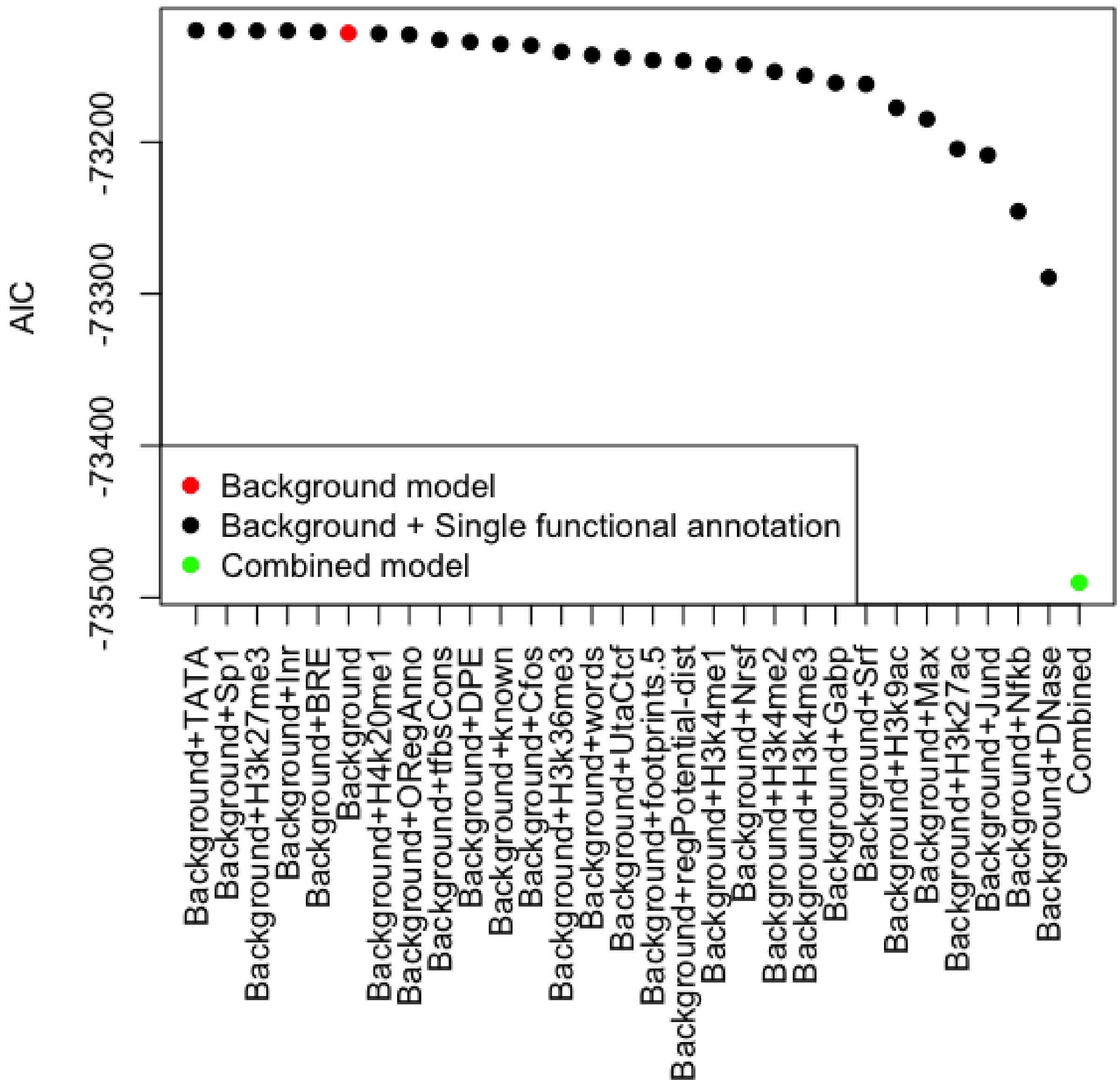

Figure S8: AIC values for all models in our analysis.

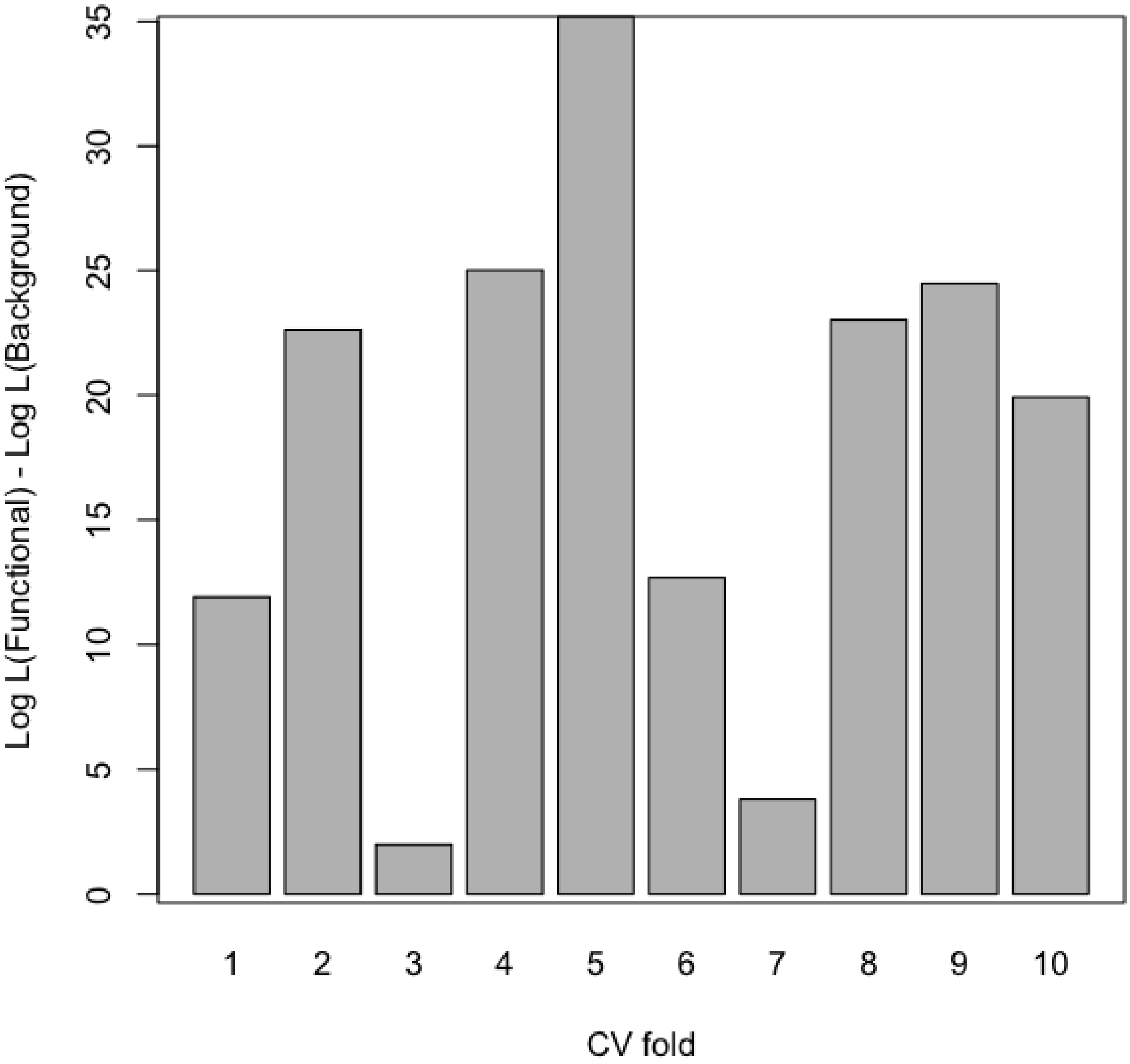

Figure S9: 10-fold cross validation results. The height of the bar represents the difference in log likelihood between combined and background models for each of the 10 held out test data sets. See the "Cross-validation" section of the Supplement for further explanation.

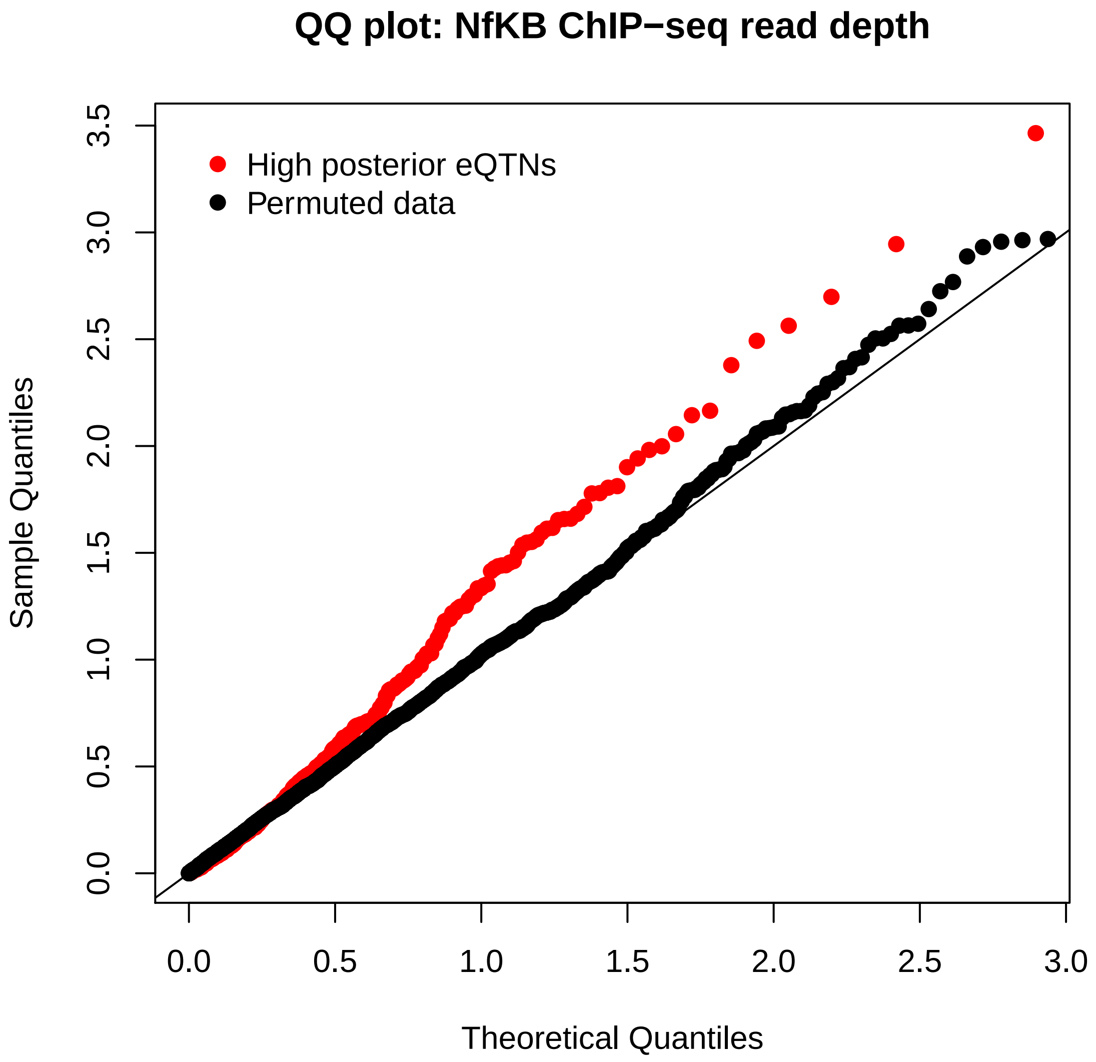

Figure S10: QQ plot showing P-values of for association with NF-κB binding, using the data of [9]. P-values are shown for high-posterior eQTNs (posterior prob> 0.5) and for permuted data, where ChIP-seq read depth was permuted randomly with respect to genotype, 5000 times.

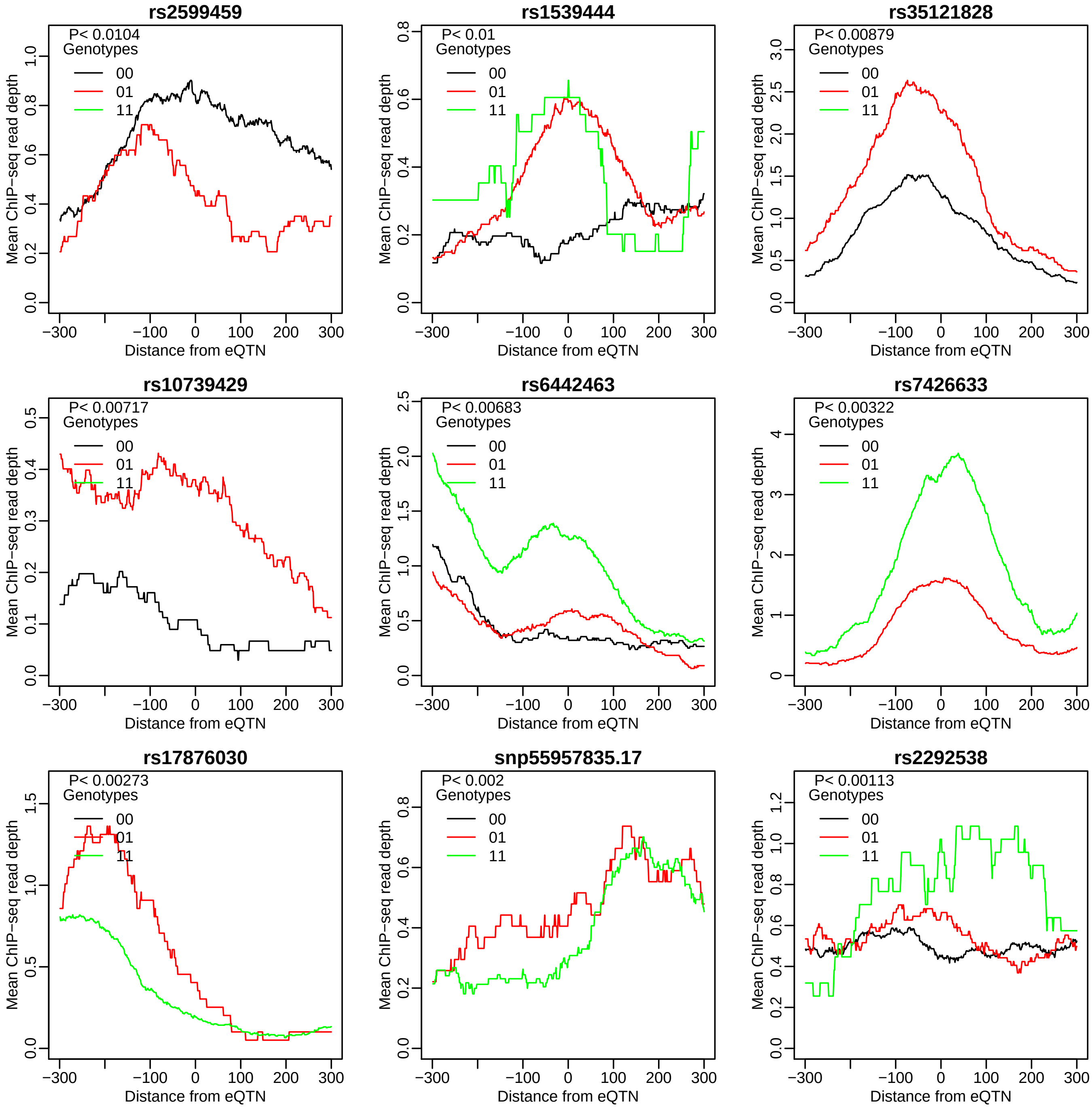

Figure S11: Examples of high posterior eQTNs that are also significantly correlated with variation in NfκB binding in 10 individuals. ChIP-seq read depth was smoothed using an 11bp Gaussian kernel, and averaged across each genotype group. P-values are for linear regression of ChIP-seq read depth in a 100bp window around the eQTN on individual genotype at that SNP.

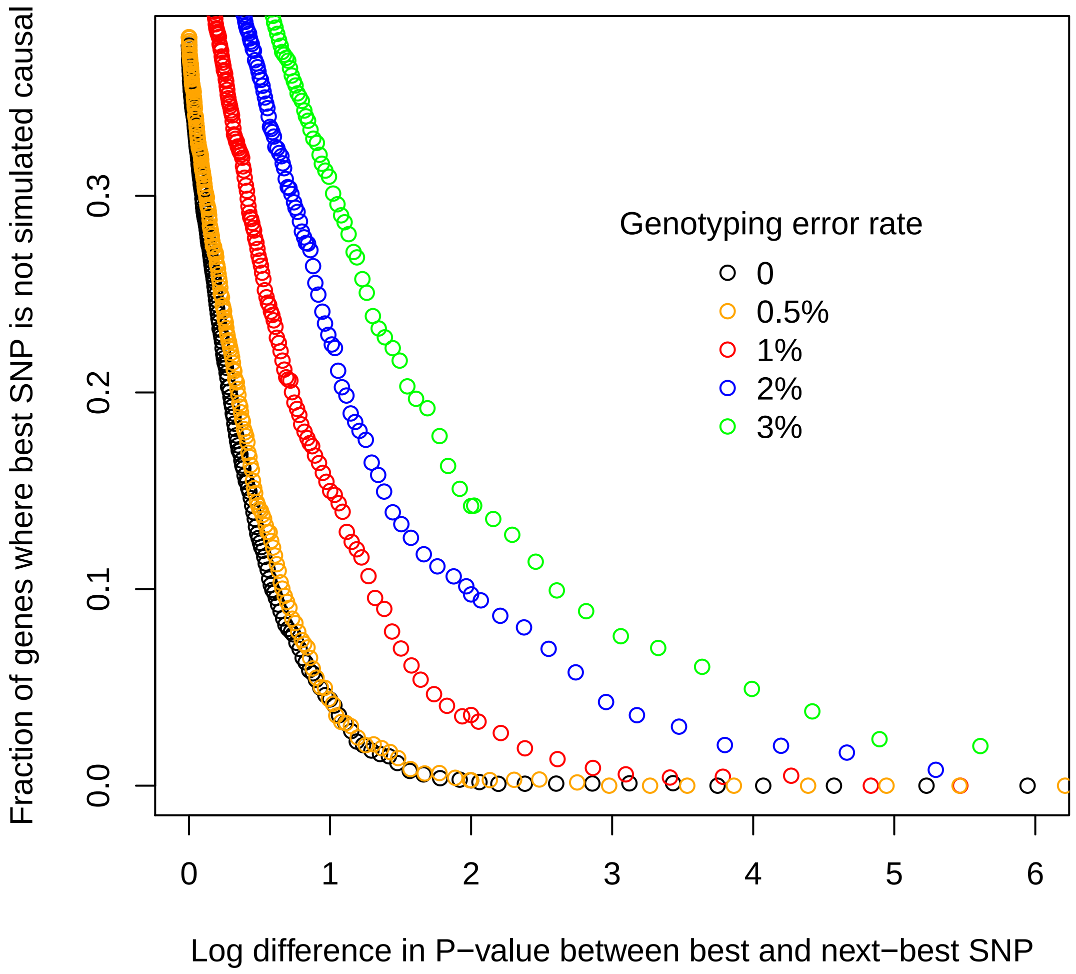

Figure S12: Effect of different P-value criteria on selection of non-causal SNPs in Monte-Carlo simulations. In each simulated replicate we selected SNPs that had P$< 5 \times 10^{-8}$ for association with expression and a variable difference in P-value between the best and next best SNP, shown on the X axis. The Y axis shows the rate at which the given criteria resulted in the selection of a non-causal SNP. Results are shown for a range of genotype error rates.

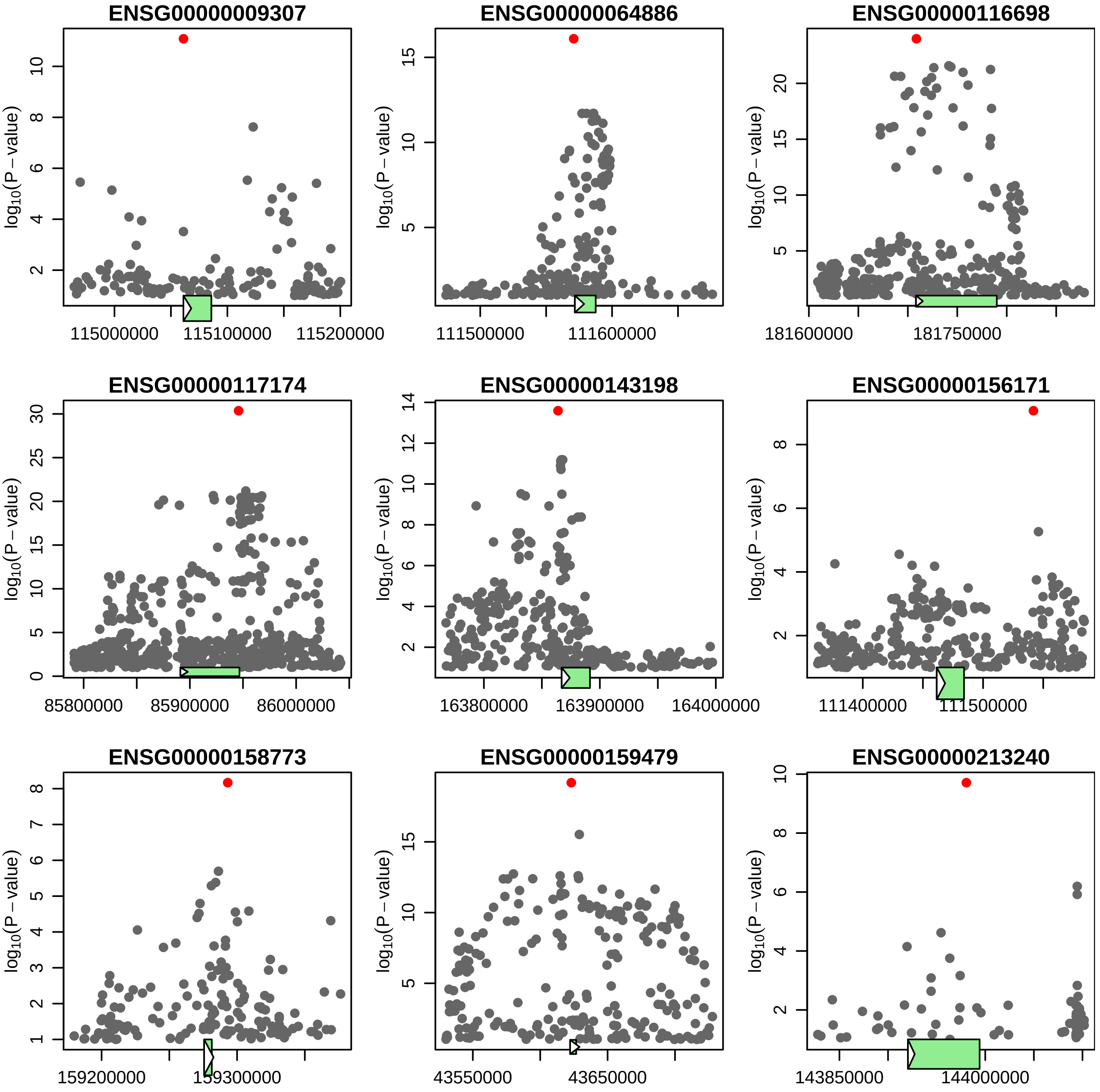

Figure S13: Nine examples of the 100 genes which made up our cross validation set. The candidate eQTN, whose location was predicted using the prior, is highlighted in red.

Supplementary Tables

Location	Annotation	No. SNPs	%eQTNs
**All**	DNase1	70588	19.9
	H3K27ac	98815	20.9
	H3K4me1	119022	9.0
	H3K4me2	125168	19.0
	H3K4me3	81558	18.5
	H3K9ac	74465	19.0
	H3K36me3	237854	7.6
	All	304219	41.8
**Upstream (>5kb from TSS)**	DNase1	36270	16.7
	H3K27ac	49522	28.1
	H3K4me1	58694	14.8
	H3K4me2	62318	22.9
	H3K4me3	40167	13.7
	H3K9ac	37623	16.2
	H3K36me3	28872	1.7
	All	147018	39.1

Table S1: Numbers of SNPs and the percentage of the estimated number of eQTNs in open chromatin and histone-modified regions. The percentage of eQTNs in each annotation was estimated by summing over the posteriors of all SNPs in that annotation, for all genes that had a high (>0.95) posterior probability of having an eQTL. Upstream of the TSS, percentages are relative to the total number of eQTNs occurring in the upstream region (~620). The fraction of eQTNs in the repressive marks H3K27me3 and H4K20me1 was estimated at close to zero and is not shown.

Clusters of TFs with similar motifs

Cluster Name	TRANSFAC/JASPAR Accession	Factor Names
**E-Box Motif binding factors**	M00118	c-Myc:Max
	M00119	Max
	M00121	USF
	M00182	GBP
	M00187	USF
	M00220	SREBP-1
	M00236	Arnt
	M00303	CBF1
	M00366	EmBP-1
	M00367	HBP-1a
	M00368	CPRF-1
	M00369	TAF-1
	M00370	CPRF-3
	M00375	TGA1b
	M00435	PIF3
	M00440	CG1
	M00441	GBF
	M00442	ABF
	M00539	Arnt
	M00615	c-Myc:Max

	M00796	USF
	M00942	CPRF-1
	M00943	TAF-1
	M00944	CPRF-3
	M00946	TGA1b
	M00985	Stra13
	M01034	Ebox
	M01116	CLOCK:BMAL
	M01145	c-Myc
	MA0058	MAX
	MA0059	MYC-MAX
**ETS family**	M00007	Elk-1
	M00016	E74A
	M00025	Elk-1
	M00032	c-Ets-1(p54)
	M00108	NRF-2/GABP
	M00339	c-Ets-1
	M00340	c-Ets-2
	M00341	GABP
	M00678	Tel-2
	M00771	Ets
	M01078	c-Ets-1
	M01163	Elk-1
	M01165	Elk-1
	M01167	SAP-1a
	M01203	PU1
	M01204	SPI-B
	M01208	FLI1
	MA0062	GABPA
	MA0076	ELK4
**Interferon Response**	M00063	IRF-2
	M00258	ISGF-3
	M00699	ICSBP
	M01066	BLIMP1
	MA0050	IRF1
	MA0051	IRF2
	MA0137	STAT1
**NF-κB**	M00051	NF-kappaB
	M00052	NF-kappaB
	M00053	c-Rel
	M00054	NF-kappaB
	M00194	NF-kappaB
	M00208	NF-kappaB
	M00774	NF-kappaB
	M01223	P50:P50
	M01224	P50:RELA-P65
	MA0022	dl_1

	MA0023	Dl_
	MA0061	NF-kappaB
	MA0101	REL
	MA0107	RELA
**CTCF**	M01200	CTCF
**Myocyte Enhancer Factor**	M00026	RSRFC4
	M00231	MEF-2
	M00232	MEF-2
	M00407	RSRFC4
	M00941	MEF-2
	MA0052	MEF2A
**FACB**	M00388	FACB
	M00390	FACB
**Early B-Cell Factor**	M00261	Olf-1
**AP-1**	M00037	NF-E2
	M00038	GCN4
	M00188	AP-1
	M00199	AP-1
	M00204	GCN4
	M00490	Bach2
	M00495	Bach1
	M00517	AP-1
	M00821	Nrf-2
	M00983	MAF
**PAX5**	M00143	Pax-5
	MA0014	Pax5
**Sp1**	M00196	Sp1
	M00255	GC
	M00491	MAZR
	M00649	MAZ
	M00807	Egr
	M00931	Sp1
	M00932	Sp1
	M00933	Sp1
	M00982	KROX
	M01068	UF1H3BETA
	M01122	ZNF219
	M01175	CKROX
	MA0079	SP1
**PU.1**	M00046	GCR1
	M00658	PU.1
**LMAF**	M01139	LMAF
**Serum Response Factor**	M00186	SRF
	M00215	SRF
	M00922	SRF
	M01007	SRF

	MA0083	SRF
**Oct1**	M00342<br>M00795	Oct-1<br>Octamer
**ESE1**<br>**PacC**<br>**E2A**	M01214<br>M00247<br>M00804	ESE1<br>PacC<br>E2A
**NHLH1**	M00058<br>M00068<br>MA0048	HEN1<br>HEN1<br>NHLH1
**Lmo2**	M00277<br>M00414	Lmo2<br>AREB6
**NFY**	M00185<br>M00209<br>M00254<br>M00287<br>M00288<br>M00309<br>M00687<br>M00775<br>MA0060	NF-Y<br>NF-Y<br>CCAAT<br>NF-Y<br>HAP2/3/4<br>ACAAT<br>alpha-CP1<br>NF-Y<br>NFYA
**Muscle**	M00321<br>M00323<br>M00324	Muscle<br>Muscle<br>Muscle
**PEBP**	M00722<br>M00769<br>M00984	core-binding<br>AML<br>PEBP
**SP1:SP3**	M00171<br>M00923<br>M01219	Adf-1<br>Adf-1<br>SP1:SP3
**AP-4**	M00005<br>M00698	AP-4<br>HEB
**HNF4**	M00411<br>M00764<br>M01031<br>M01036	HNF4alpha1<br>HNF4<br>HNF4<br>COUPTF

Table S2: Transcription factor names and TRANSFAC or JASPAR accessions for clusters of DNase footprints.

References

[1] Veyrieras JB, Kudaravalli S, Kim SY, Dermitzakis ET, Gilad Y, Stephens M, Pritchard JK: **High-Resolution Mapping of Expression-QTLs Yields Insight into Human Gene Regulation**. *Plos Genetics* 2008, **4**.

[2] Servin B, Stephens M: **Imputation-based analysis of association studies: Candidate regions and quantitative traits**. *Plos Genetics* 2007, **3**:1296–1308.

[3] Kamatani Y, Matsuda K, Okada Y, Kubo M, Hosono N, Daigo Y, Nakamura Y, Kamatani N: **Genome-wide association study of hematological and biochemical traits in a Japanese population**. *Nature Genetics* 2010, **42**:210–U25.

[4] Hindorff LA, Junkins HA, Mehta JP, Hall P, Manolio TA: **A Catalog of Published Genome-Wide Association Studies** 2010, [http://www.genome.gov/gwastudies].

[5] Kolbe D, Taylor J, Elnitski L, Eswara P, Li J, Miller W, Hardison R, Chiaromonte F: **Regulatory potential scores from genome-wide three-way alignments of human, mouse, and rat**. *Genome Research* 2004, **14**:700–707.

[6] Siepel A, Bejerano G, Pedersen JS, Hinrichs AS, Hou MM, Rosenbloom K, Clawson H, Spieth J, Hillier LW, Richards S, Weinstock GM, Wilson RK, Gibbs RA, Kent WJ, Miller W, Haussler D: **Evolutionarily conserved elements in vertebrate, insect, worm, and yeast genomes**. *Genome Res.* 2005, **15**:1034–1050.

[7] Pollard KS, Hubisz MJ, Rosenbloom KR, Siepel A: **Detection of nonneutral substitution rates on mammalian phylogenies**. *Genome Research* 2010, **20**:110–121.

[8] Xie XH, Rigor P, Baldi P: **MotifMap: a human genome-wide map of candidate regulatory motif sites**. *Bioinformatics* 2009, **25**:167–174.

[9] Kasowski M, Grubert F, Heffelfinger C, Hariharan M, Asabere A, Waszak SM, Habegger L, Rozowsky J, Shi MY, Urban AE, Hong MY, Karczewski KJ, Huber W, Weissman SM, Gerstein MB, Korbel JO, Snyder M: **Variation in Transcription Factor Binding Among Humans**. *Science* 2010, **328**:232–235.
